# Supplementary material for: Photoswitchable polyynes for multiplexed stimulated Raman scattering microscopy with reversible light control
Source: Nat Commun. 2024 Mar 22;15:2578. doi: 10.1038/s41467-024-46904-6 (PMC10959996; doi:10.1038/s41467-024-46904-6)
Supplement: Supplementary file 1 — Supplementary Information [file 41467_2024_46904_MOESM1_ESM.pdf]

Supplementary Information

Photoswitchable polyynes for multiplexed stimulated Raman scattering microscopy with reversible light control

Supplementary Figures

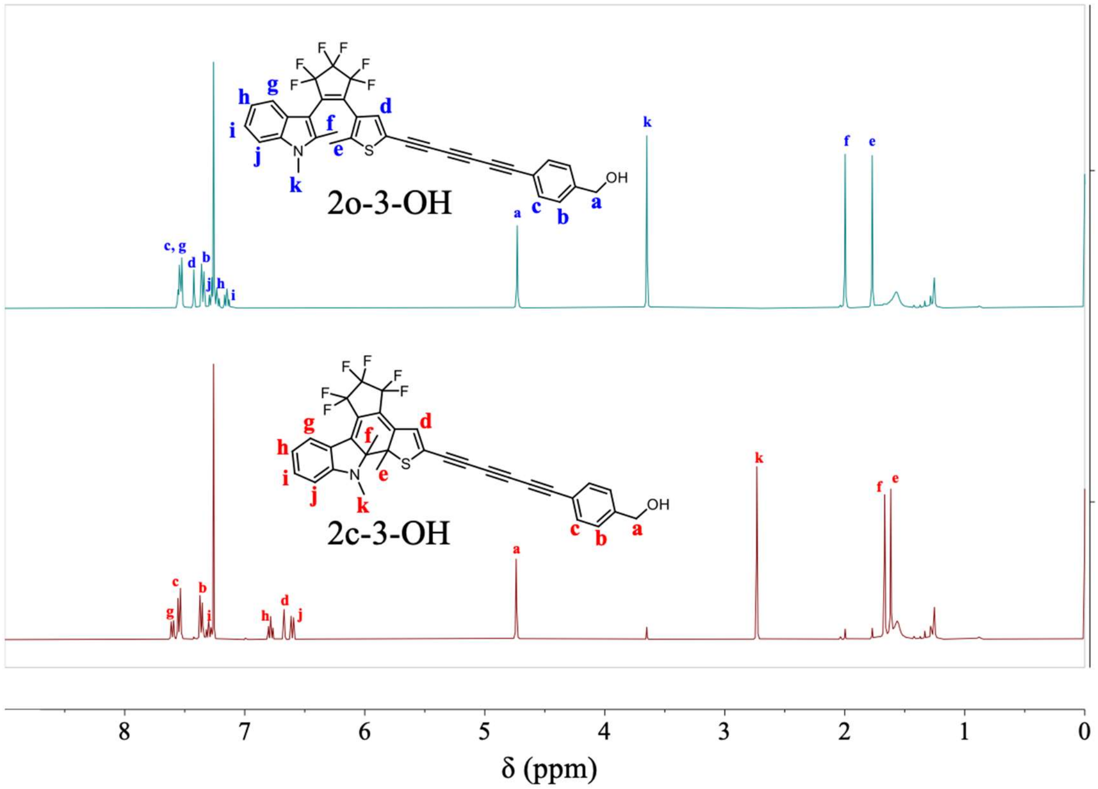

Supplementary Fig. 1. ^{1}H NMR spectra of 2-3-OH (open and closed forms) in $CDCl_3$. After photocyclization from the open to closed form, three methyl protons H_k, H_f, H_e of 2o-3-OH were all upfield shifted (H_k: $\Delta\delta = 0.92$ ppm, H_f: $\Delta\delta = 0.33$ ppm, H_e: $\Delta\delta = 0.15$ ppm). The conversion yield was calculated to be 91% from H_k, 93% from H_f and 91% from H_e by ^{1}H NMR integration of methyl groups in two forms.

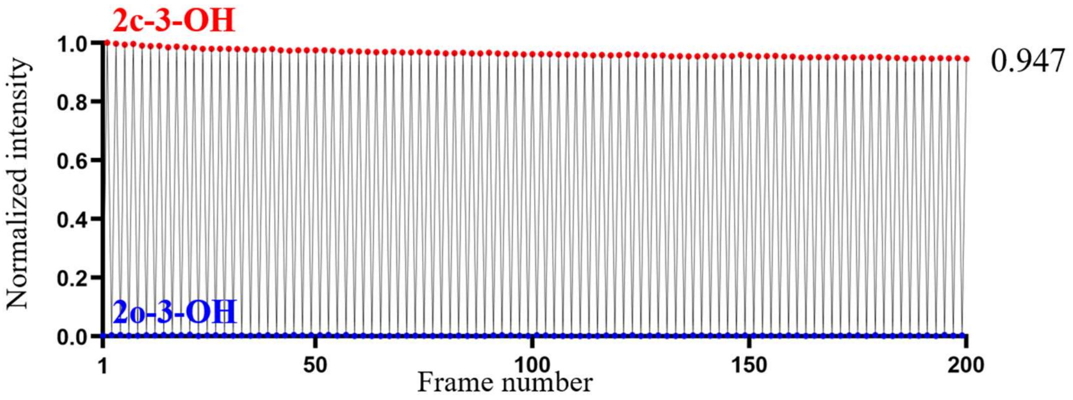

Supplementary Fig. 2. Reversible photoswitching and photo-fatigue resistance of 2-3-OH under alternating 405 nm and 640 nm irradiation of the entire 20 μM DMSO solution. Absorption was measured at 675 nm to be steady with little decay over 100 cycles.

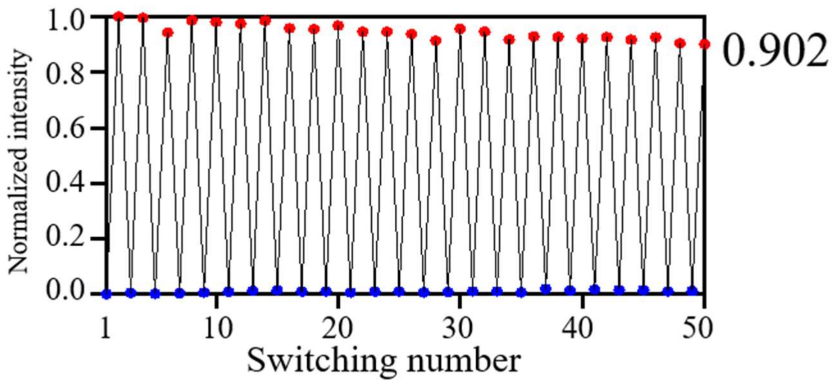

Supplementary Fig. 3. Reversible photoswitching and photo-fatigue resistance of 2-3-OH by SRS readout. Under alternating 405 nm and 640 nm irradiation of the entire 5 mM DMSO solution, SRS signal was measured at 2147 cm^{-1} with over 90% intensity after 50 times of photoswitching.

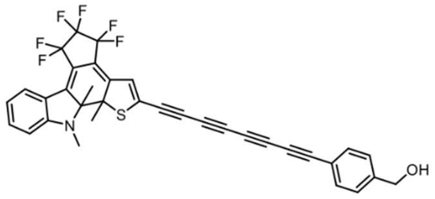

2c-4-OH

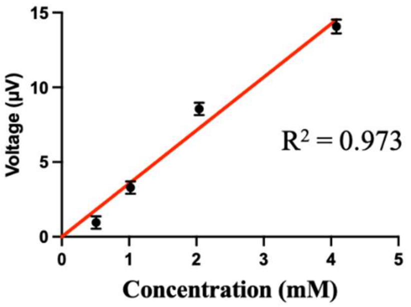

Supplementary Fig. 4. Chemical structure and linear concentration dependence of 2c-4-OH with SRS intensity (mean±s.d.).

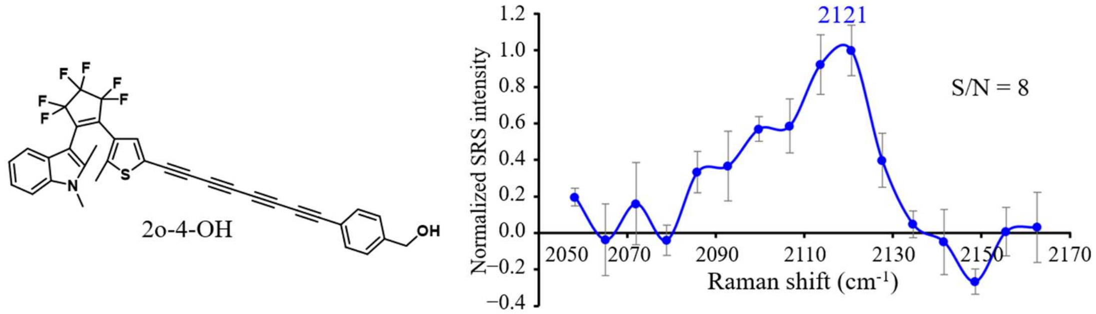

Supplementary Fig. 5. SRS spectrum of 10 μM 2o-4-OH in DMSO solution. The spectrum was measured under 1 ms time constant with a signal to noise ratio of 8. The curve was averaged over 3 measurements (mean±s.d.).

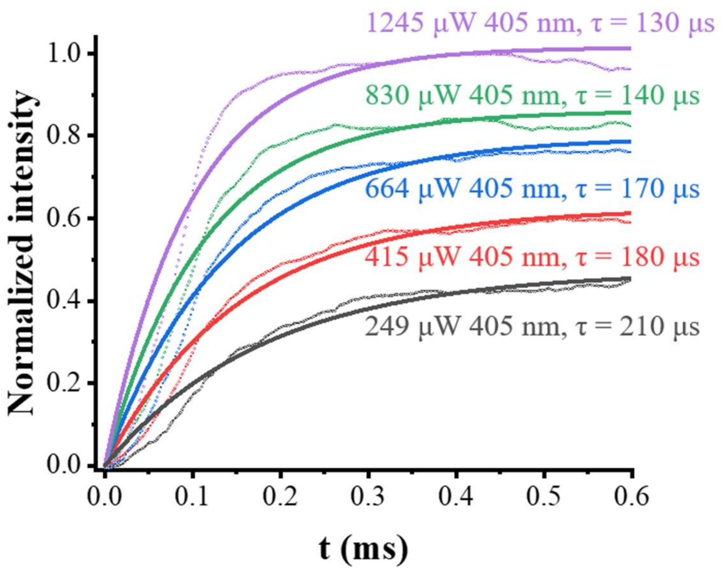

Supplementary Fig. 6. Photoswitching kinetics of 2-3-OH under 405 nm irradiations of different power. Microwatt-level power of 405 nm light can switch on 2-3-OH within 200 μs. Each curve was averaged over 10 independent measurements.

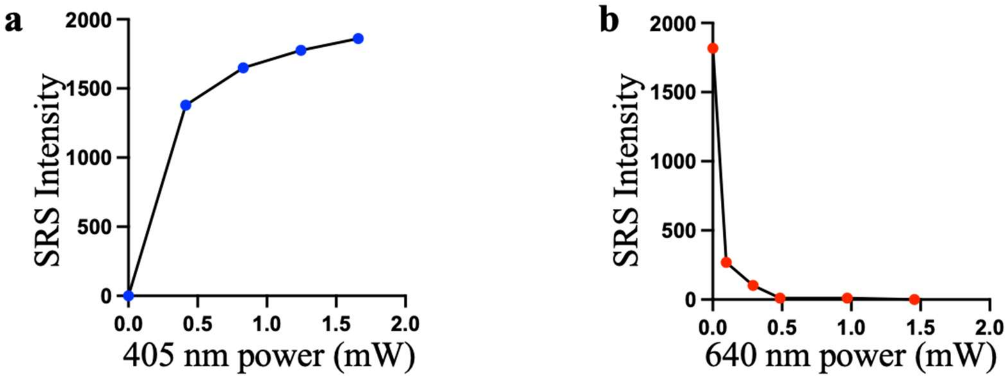

Supplementary Fig. 7. Photoswitching power dependence of 2-3-OH under 405 nm (a, on-switching) or 640 nm (b, off-switching) irradiations (40 μs pixel dwell time and 256×256 pixel size). <1 mW of 405 nm and 640 nm illumination were sufficient to achieve high photo-conversions of 2-3-OH for SRS imaging.

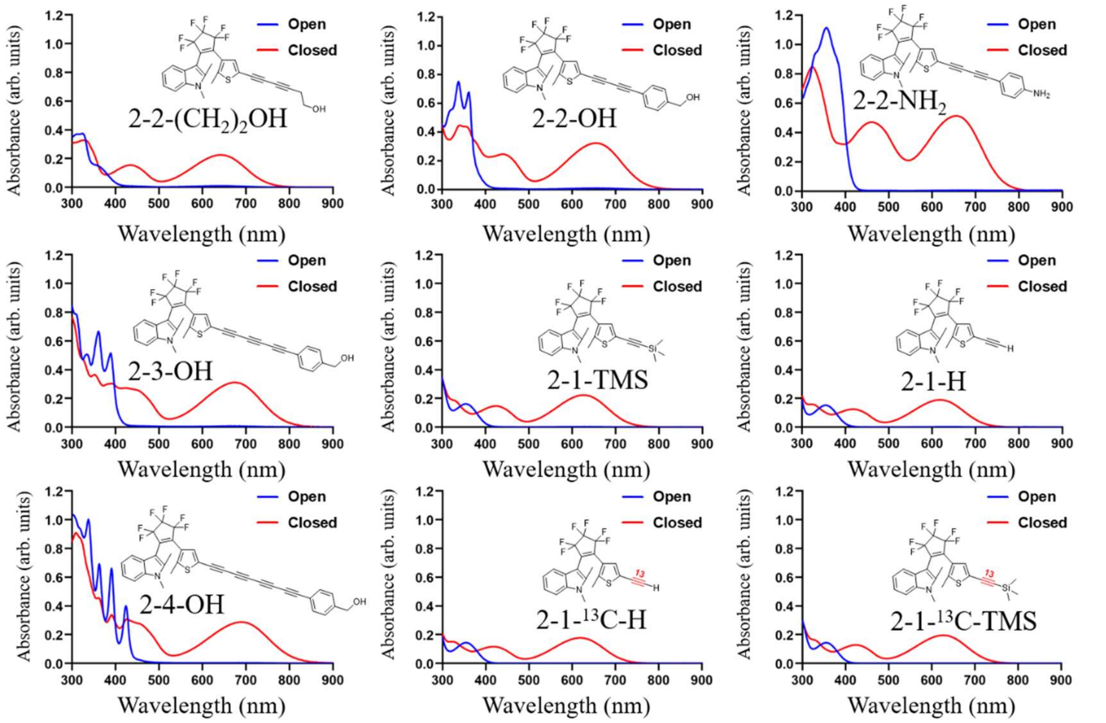

Supplementary Fig. 8. UV-Vis absorption spectra of Carbow-switch molecules in both open and closed forms. All molecules are measured at 20 μM in DMSO, except 2-4-OH measured at 16 μM in DMSO.

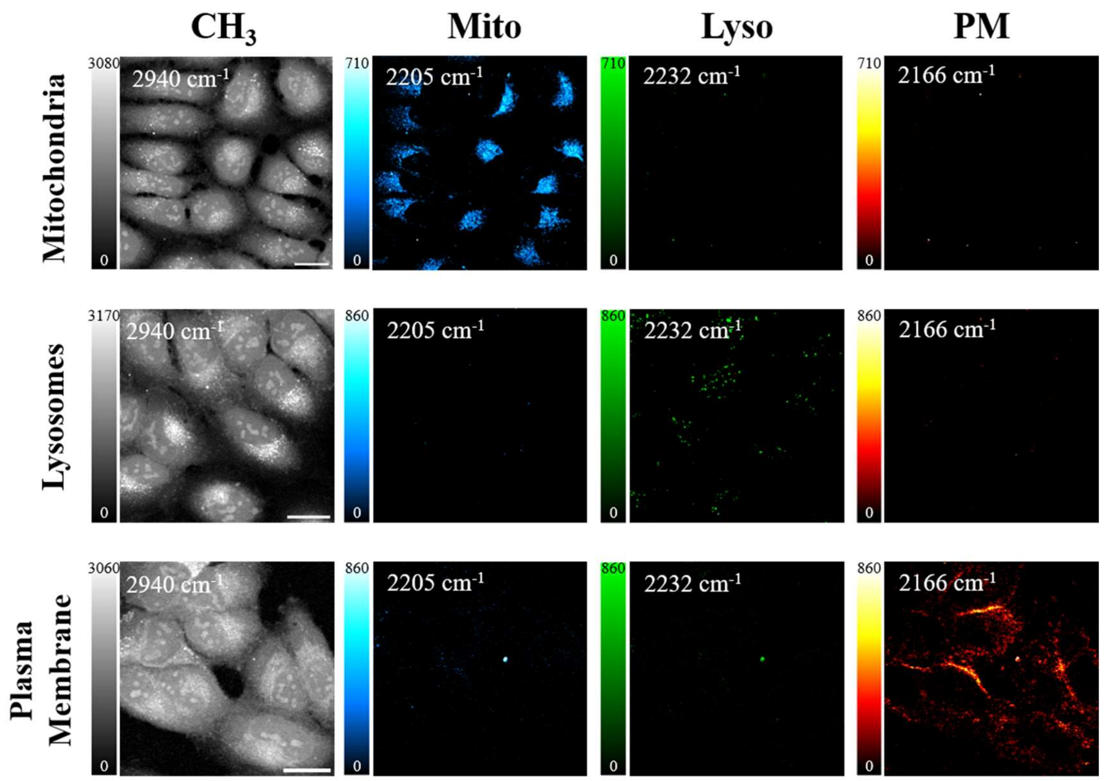

Supplementary Fig. 9. SRS imaging of organelle-targeted Carbow-switch at three distinct frequencies (2205 cm^{-1} for Mito-switch, 2232 cm^{-1} for Lyso-switch, and 2166 cm^{-1} for PM-switch). Little spectral crosstalk was observed among organelle-targeted Carbow-switch, demonstrating high molecular vibrational specificity. Protein CH_3 (2940 cm^{-1}) were imaged to show the cell morphology. Scale bar: 20 μm. Experiments were repeated three times independently with similar results.

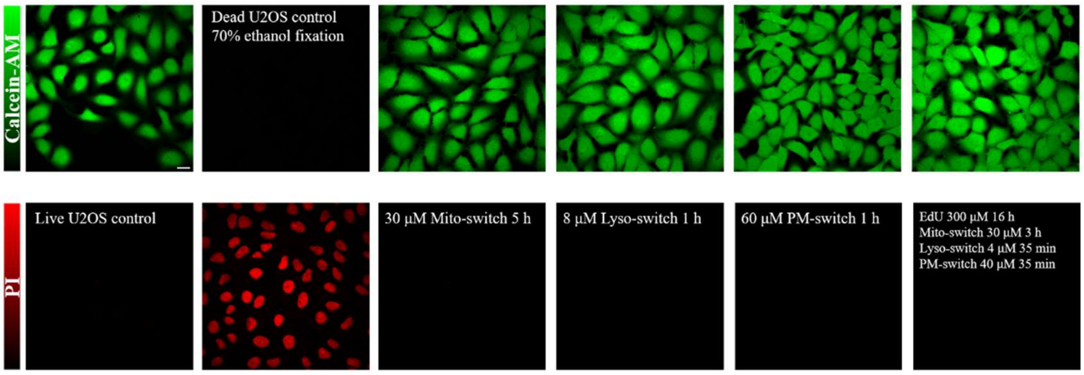

Supplementary Fig. 10. Viability assay of organelle-targeted Carbow-switch in live U2OS cells. Carbow-switch probes were incubated at the indicated concentrations and time before two-color fluorescence imaging. Multiplexed Carbow-switch labeling exihibited little cytotoxicity in live cells (Calcein-AM in green as live cell marker and PI in red as dead cell marker). Scale bar: 20 μm. Experiments were repeated at least three times independently with similar results.

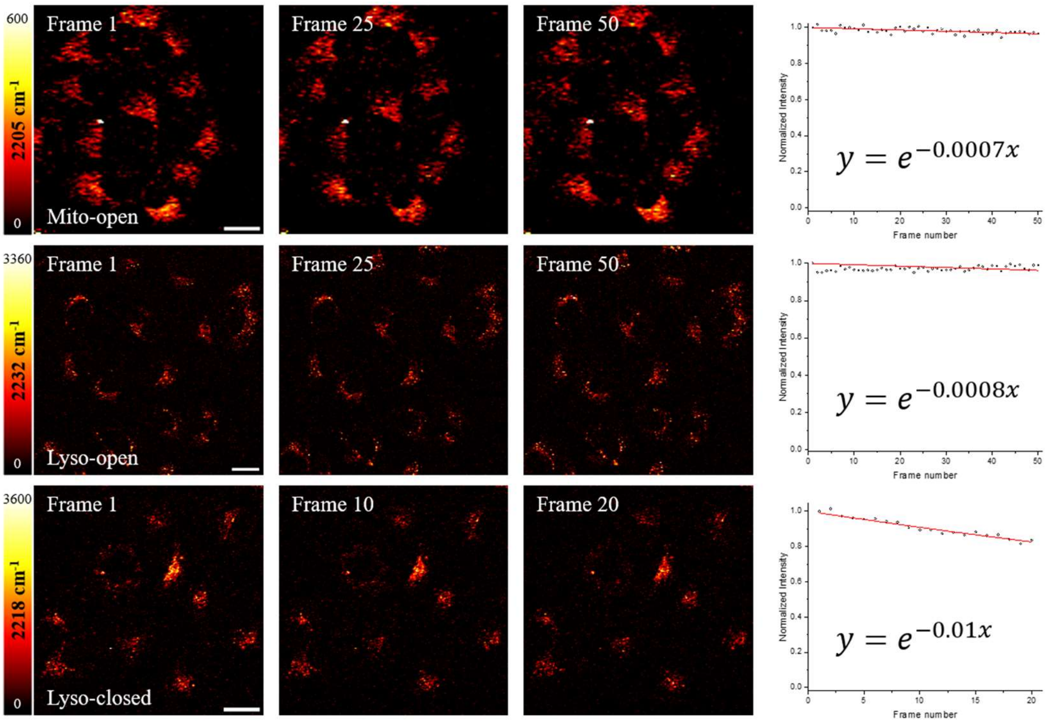

Supplementary Fig. 11. Photostability characterization of Mito-switch and Lyso-switch by live-cell continous SRS imaging. SRS intensity of both Mito-switch and Lyso-switch in the open form stayed steady over 95% after continuous 50-frame imaging. In the closed form, over 80% signal of Lyso-switch remained after continuous 20-frame imaging. Scale bar: 20 μm. Experiments were repeated three times independently with similar results.

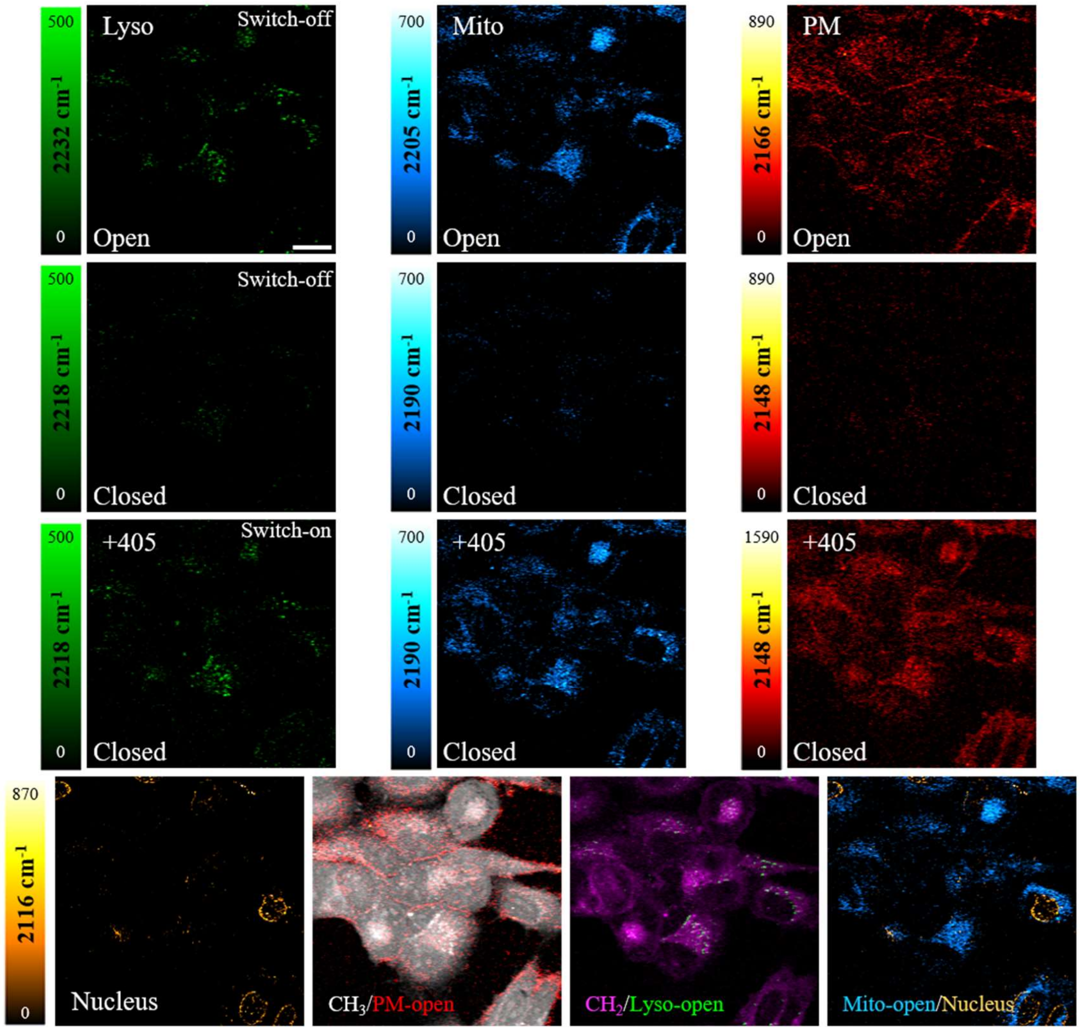

Supplementary Fig. 12. Live-cell multiplexed SRS imaging of Carbow-switch at both open and closed forms. 9-color SRS imaging of organelles in live cells was performed at both photoswitching forms of Carbow-switch, including lysosomes Lyso-switch (open and closed frequencies, 2232 and 2218 cm^{-1}), mitochondria Mito-switch (open and closed frequencies, 2205 and 2190 cm^{-1}), plasma membrane PM-switch (open and closed frequencies, 2166 and 2148 cm^{-1}), nucleus (EdU, 2116 cm^{-1}), total protein CH_3 (2940 cm^{-1}), and total lipids CH_2 (2845 cm^{-1}). The switch-off and switch-on images corresponded to the open and closed forms of Carbow-switch, respectively. Overlay of two images were shown for the same set of cells. Scale bar: 20 μm. Experiments were repeated three times independently with similar results.

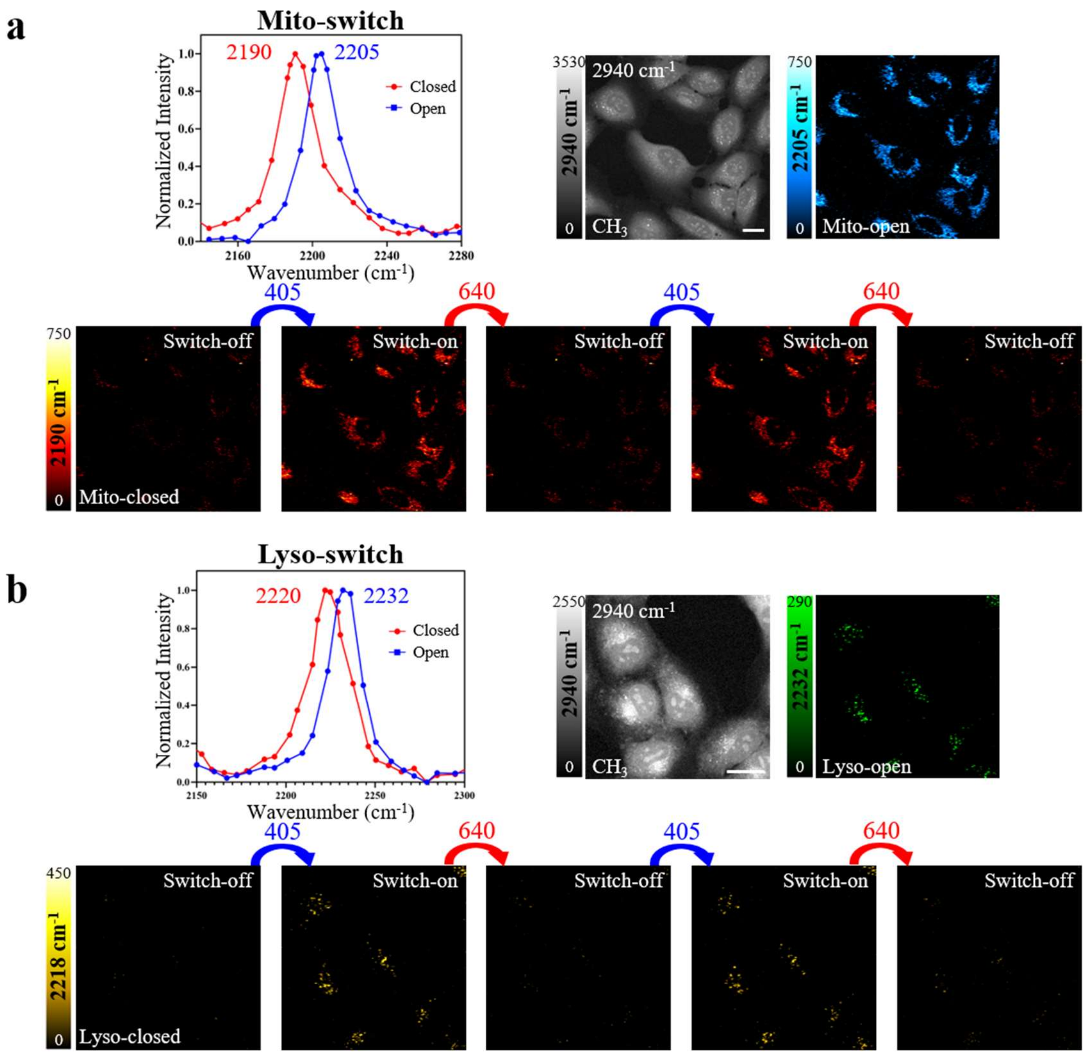

Supplementary Fig. 13. Photoswitchable SRS spectra and reversible imaging of Mito-switch (**a**) and Lyso-switch (**b**) in live cells. The SRS signal of mitochondria (2190 cm^{-1} of Mito-closed channel) and lysosomes (2218 cm^{-1} of Lyso-closed channel) can be efficiently switched on by 405 nm light and switched off by 640 nm light in live cells with low background and high contrast. Protein CH_3 (2940 cm^{-1}) were imaged to show the cell morphology. Mito-open (2205 cm^{-1}) and Lyso-open (2232 cm^{-1}) were imaged to show the distribution before photoswitching. Scale bar: 20 μm. Experiments were repeated three times independently with similar results.

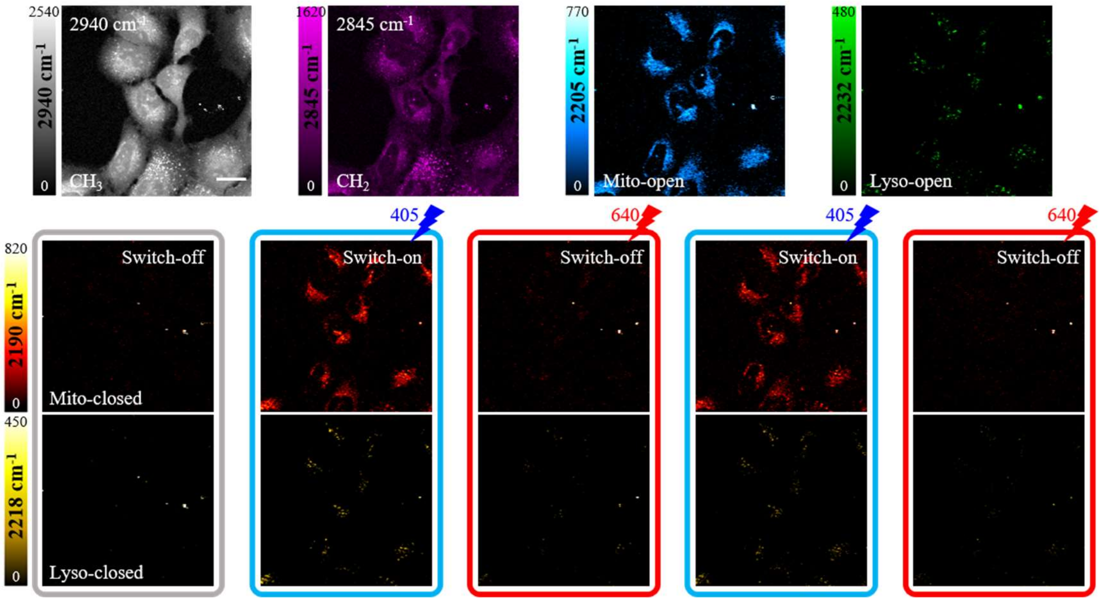

Supplementary Fig. 14. Multi-color reversible SRS imaging of Mito-switch and Lyso-switch in live cells. The SRS signal of mitochondria (2190 cm^{-1} of Mito-closed channel) and lysosomes (2218 cm^{-1} of Lyso-closed channel) were reversibly switched on and off over two cycles. Protein CH_3 (2940 cm^{-1}) and Lipid CH_2 (2845 cm^{-1}) were imaged to show the cell morphology. Mito-open (2205 cm^{-1}) and Lyso-open (2232 cm^{-1}) were imaged to show the distribution before photoswitching. Scale bar: 20 μm. Experiments were repeated three times independently with similar results.

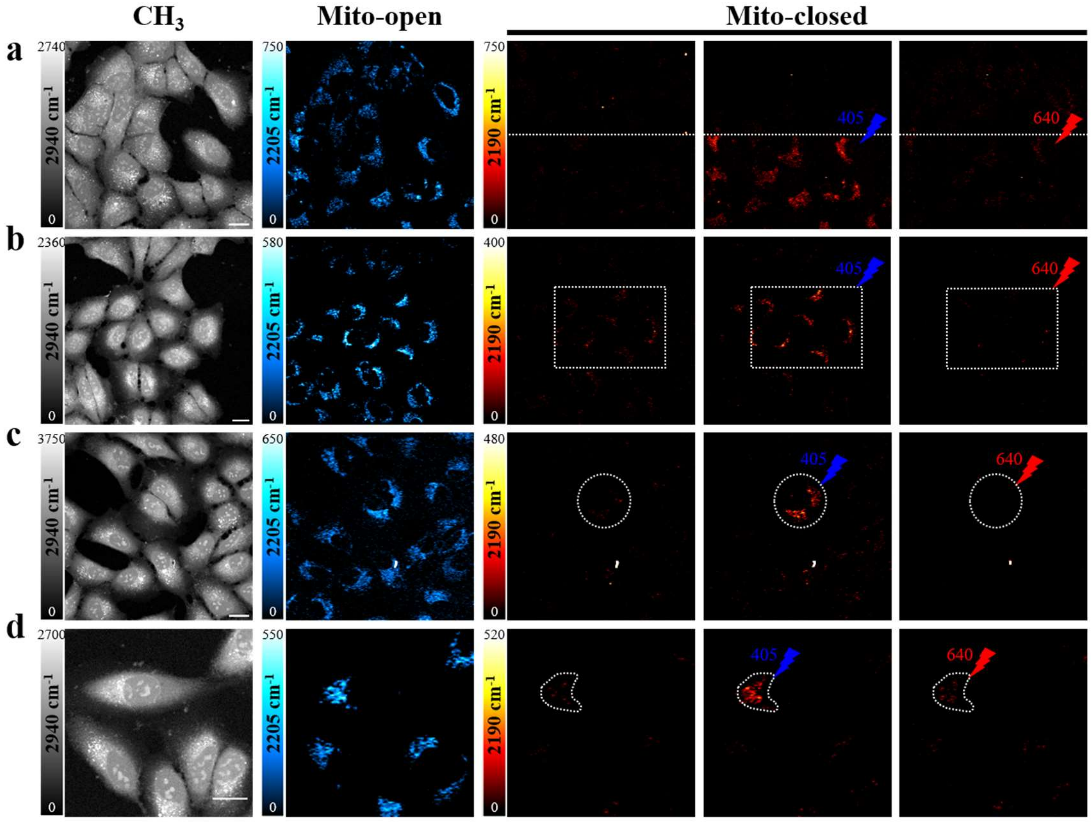

Supplementary Fig. 15. Region-selective SRS imaging of Mito-switch (2190 cm^{-1} of the Mito-closed channel) in live cells with different modes. (**a**) half field of view; (**b**) rectangular; (**c**) circular; (**d**) single cell. Selected regions of 405 and 640 nm irradiation are indicated by white dashed lines. Protein CH_3 channels (2940 cm^{-1}) were imaged to show the cell morphology and Mito-open channels (2205 cm^{-1}) were imaged to show the total distribution of mitochondria before photoswitching. Scale bar: 20 μm. Experiments were repeated at least three times independently with similar results.

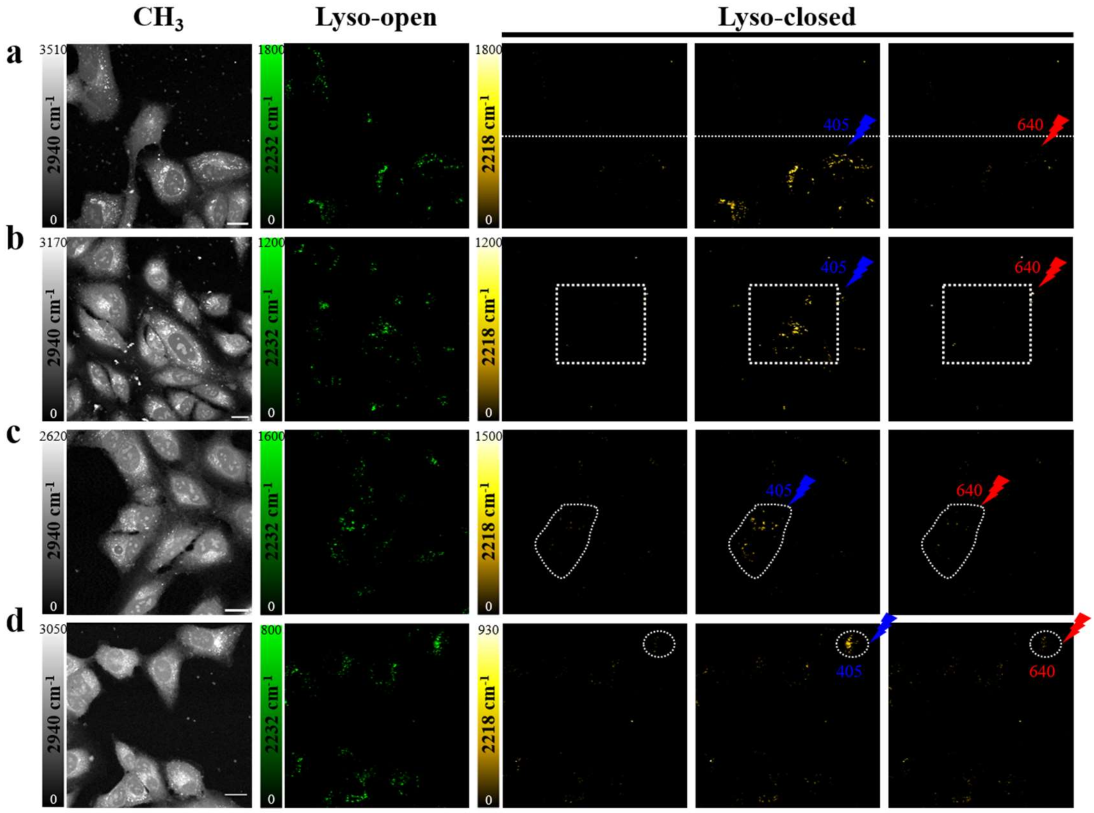

Supplementary Fig. 16. Region-selective SRS imaging of Lyso-switch (2218 cm^{-1} of the Lyso-closed channel) in live cells with different modes. (**a**) half field of view; (**b**) square; (**c**) irregular shape; (**d**) single cell. Selected regions of 405 and 640 nm irradiations were indicated by white dashed lines. Protein CH_3 channels (2940 cm^{-1}) were imaged to show the cell morphology and Lyso-open channels (2232 cm^{-1}) were imaged to show the total distribution of lysosomes before photoswitching. Scale bar: 20 μm. Experiments were repeated at least three times independently with similar results.

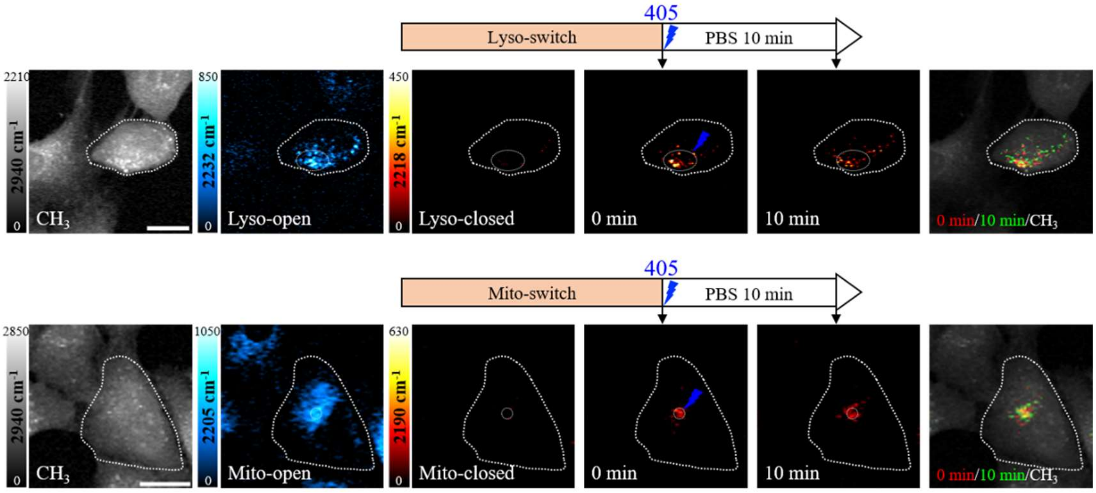

Supplementary Fig. 17. Subcellular photoswitchable SRS imaging and time-lapse tracking of lysosomes and mitochondria inside living cells. In U2OS cells, subpopulations of lysosomes and mitochondria were selectively switched on by 405 nm irradiation (white dashed circles) and imaged at Lyso-closed channel (2218 cm^{-1}) and Mito-closed (2190 cm^{-1}) channels. Time-lapse SRS imaging showed fast organelle dynamics inside single cells after 10 min in PBS. Overlay image of lysosomes and mitochondria at 0 min (red) and 10 min (green) showed organelle movements at two time points. Protein CH_3 channels (2940 cm^{-1}) were imaged to show the cell morphology. Lyso-open channel (2232 cm^{-1}) and Mito-open channel (2205 cm^{-1}) were imaged to show the total distribution of organelles before photoswitching. Scale bar: 20 μm. Experiments were repeated three times independently with similar results.

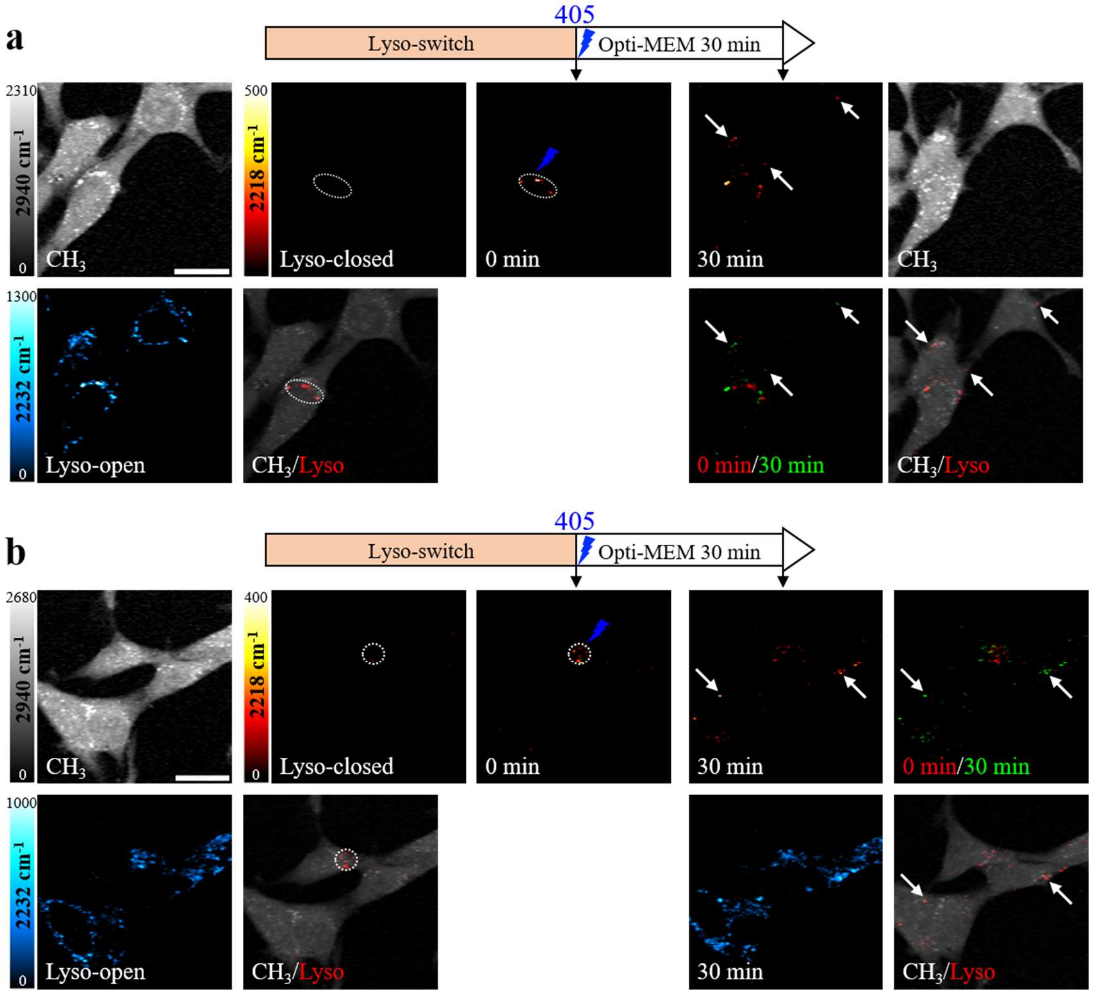

Supplementary Fig. 18. Photoswitchable SRS imaging and tracking of lysosomes among MEF cells. In MEF cells cultured on fibronectin-coated coverslips, lysosomes were selectively switched on in one cell by 405 nm irradiation (white dashed lines) and imaged at Lyso-closed channels (2218 cm^{-1}). Time-lapse SRS imaging of selected lysosomes showed large movements from one cell to the neighboring cells (white arrows) after 30 min. Overlay image of lysosomes at 0 min (red) and 30 min (green) suggested the intercellular trafficking of lysosomes at two time points. Protein CH_3 channels (2940 cm^{-1}) were imaged to show the cell morphology. Lyso-open channels (2232 cm^{-1}) were imaged to show the distribution of lysosomes. Scale bar: 20 μm. Experiments were repeated three times independently with similar results.

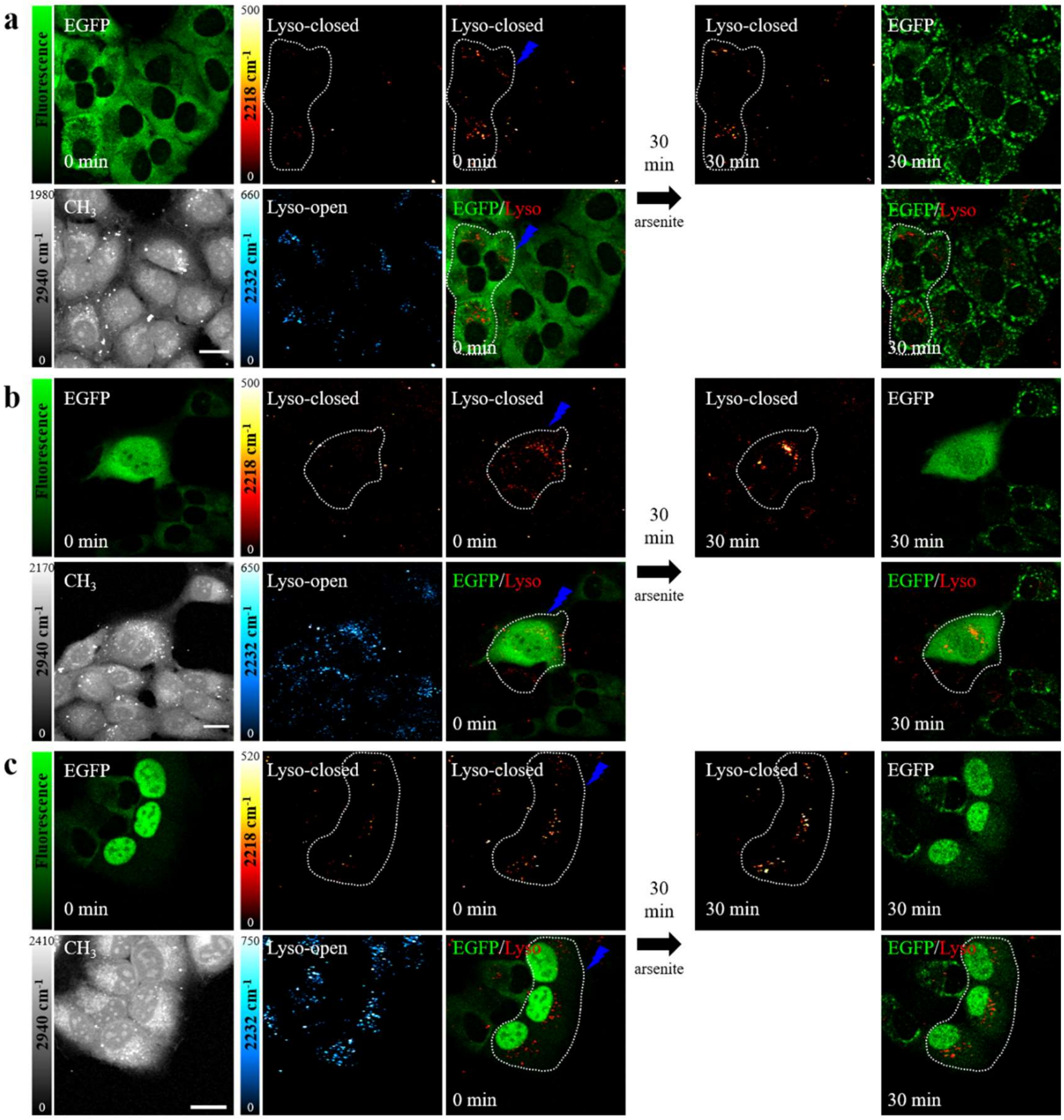

Supplementary Fig. 19. Time-lapse fluorescence and SRS imaging of selective lysosome dynamics in live cells during stress granule formation. (**a**) In cells with EGFP-ATXN2 expression mainly in the cytoplasm, many stress granules formed under 30 min sodium arsenite treament and the distribution of lysosomes remained scattered. (**b**) In cells with EGFP-ATXN2 expression in both the cytoplasm and nucleus, little stress granules were formed and lysosomes showed strong clustering near the nucleus. (**c**) In cells with EGFP-ATXN2 expression only in the nucleus, little stress granules were formed and lysosomes formed clusters. Selected cells were switched on by 405 nm irradiation for photo-selective SRS imaging (white dashed lines). Protein CH_3 channels were imaged to show the cell morphology and Lyso-open channels were imaged to show the total distribution of lysosomes before photoswitching. Scale bar: 20 μm. Experiments were repeated three times independently with similar results.

Supplementary Tables

Supplementary Table 1. UV-Vis absorption spectra of asymmetric diarylethene polyynes

Compound	λ_{max} (open, nm)	ε (open, 10^4 M^{-1} cm^{-1})	λ_{max} (closed, nm)	ε (closed, 10^4 M^{-1} cm^{-1})	FWHM (closed, nm)	Conversion yield (%)
1-1	309	3.00	562	2.18	122	38
2-1	312	2.75	634	1.78	139	72
3-1	307	2.79	675	1.47	157	83
4-1	313	2.66	724	0.17[a]	203	—
3-2	337	3.30	697	1.67	166	85
3-3	359	3.83	722	1.86	178	93
3-2-NH_2	358	4.98	694	2.31	167	86
3-2-CN	350	3.43	705	1.59	172	92
2-2-$(CH_2)_2OH$	324	1.87	644	1.73	142	65
2-2-OH	362[b]	3.38	656	2.21	147	73
2-2-NH_2	357	5.75	656	2.99	151	86
2-3-OH	389[b]	2.57	675	1.72	154	91
2-1-TMS	355	0.81	628	1.45	136	77
2-4-OH	424[b]	2.19	688	2.50	162	72
2-1-H	354	0.76	618	1.23	133	77
2-1-^{13}C-TMS	355	0.72	626	1.31	136	75
2-1-^{13}C-H	354	0.72	620	1.19	134	75

The UV-Vis absoption spectra were tested in DMSO. The conversion yield was measured by ^1H NMR in $CDCl_3$.

[a] The extinct coefficient is not corrected by conversion yield.

[b] The longest wavelengths of absorption are shown.

Supplementary Table 2. SRS spectra of asymmetric diarylethene polyynes derivatives

**Compound**	**Form**	**SRS frequency (cm^{-1})**	**FWHM (cm^{-1})**	**RIE**	**Peak intensity ratio (closed/open)**	**Intensity ratio at closed frequency (closed/open)**
**2-1**	Open	**2206**	21	6.9	8.6	30
	Closed	**2194**	26	59		
**2-2-(CH_2)$_2$OH**	Open	**2232**	22	7.4	5.2	16
	Closed	**2219**	26	38		
2-2-OH	Open	2205	23	23	3.7	12
	Closed	2190	28	86		
**2-2-NH_2**	Open	**2194**	21	49	8.4	91
	Closed	**2170**	32	416		
**2-3-OH**	Open	**2166**	21	57	4.8	32
	Closed	**2147**	30	275		
2-1-TMS	Open	2145	20	2.5	5.8	12
	Closed	2136	21	15		
**2-4-OH**	Open	**2121**	20	117	4.2	14
	Closed	**2108**	29	489		
**2-1-H**	Open	**2094**	20	1.1	6.7	75
	Closed	**2081**	22	7.7		
**2-1-^{13}C-TMS[a]**	Open	**2067**	19	1.8	9.0	22
	Closed	**2055**	30	16		
**2-1-^{13}C-H[a]**	Open	**2024**	20	1.3	6.4	32
	Closed	**2011**	23	8.5		

The SRS spectra were measured in DMSO. Selected compounds shown in Figure 3 were highlighted in bold.

[a] 1900 cm^{-1} was used as off-resonance.

Supplementary Table 3. SRS spectra of organelle-targeting Carbow-switch

**Compound**	**Structure**	**SRS frequency (open, cm^{-1})**	**FWHM (open, cm^{-1})**	**SRS frequency (closed, cm^{-1})**	**FWHM (closed, cm^{-1})**
Mito-switch	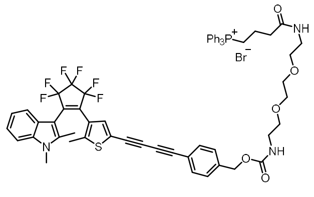	2205	23	2190	25
Lyso-switch	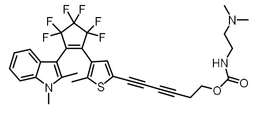	2232	22	2220	27
PM-switch	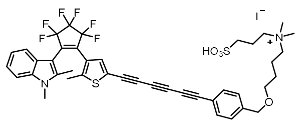	2166	26	2148	27

Supplementary Notes

General Information

All reagents and solvents were purchased and used without futher purification unless otherwise noted. DCM, DMF, PE, EA, DMSO, THF, NBS and TMSA respectively refer to dichlormethane, *N*,*N*-dimethylformamide, petroleum ether (60-90 ºC), ethyl acetate, dimethyl sulfoxide, tetrahydrofuran, *N*-bromosuccinimide and trimethylsilylacetylene. Column chromatography was performed on 200-300 mesh silica gel. TLC was measured on 0.2 mm Huanghai silica plates GF-254 and was visualized using UV light.

Nuclear magenetic resonance (NMR) spectra were measured on Bruker AVANCE III (400 MHz). NMR spetra were calibrated (^{1}H: δ 7.26 for $CDCl_3$; ^{1}H: δ 2.50 for DMSO-*d6*; ^{1}H: δ 3.31 for CD_3OD; ^{13}C: δ 77.16 for $CDCl_3$). Abbreviations were explained multiplicities: s = singlet, d = doublet, t = triplet, q = quartet, m = multiplet. High resolution mass spectra (HRMS) were obtained from LCMS-IT-TOF of SHIMADZU and ACQUITY UPLC I-Class PLUS.

Asymmetry diarylethene polyynes were composed with aromatic rings, cyclopentene and end-capped polyynes. The two step nucleophilic substitutions mediated with *n*-BuLi formed the asymmetry structure[1]. The polyynes was synthesized by using Glaser-Hay coupling and Cadiot-Chodkiewicz coupling[2].

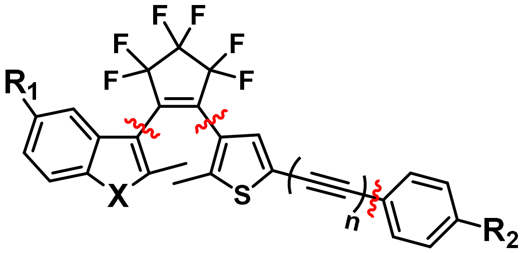

Synthesis of aryl segments

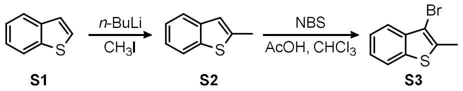

Benzo[*b*]thiophene **S1** (5.0 g, 37.26 mmol, 1 eq) was dissolved in THF (100 mL) under N_2 atmosphere and cooled to -78 ºC. *n*-Butyllithium (2.5 M, 22.4 mL, 1.5 eq) was added dropwise slowly. The reaction was stirred for 1 h at -78 ºC, then iodomethane (6.88 g, 48.44 mmol, 1.3 eq) was added and the reaction was stirred for 6 h at room temperature. The reaction was quenched by water and extracted with DCM. The organic phase was dried with anhydrous Na_2SO_4. The organic solvent was evaporated and the residue was purified with flash column chromatography (hexane) to obtain a white solid **S2** (5.1 g, 92%). ^{1}H NMR (400 MHz, $CDCl_3$) δ 7.75 (d, *J* = 7.7 Hz, 1H), 7.66 (d, *J* = 7.8 Hz, 1H), 7.31 (t, *J* = 7.4 Hz, 1H), 7.25 (t, *J* = 7.5 Hz, 1H), 6.98 (s, 1H), 2.60 (s, 3H).

NBS (7.53 g, 42.30 mmol, 1.1 eq) was added to a solution of 2-methylbenzo[*b*]thiophene **S2** (5.70 g, 38.46 mmol, 1eq) in AcOH/$CHCl_3$ (20 mL v/v = 1:1) at 0 ºC. the reaction was stirred for 4 h. The reaction was quenched by water and extracted with DCM. The organic phase was dried with anhydrous Na_2SO_4. The organic solvent was evaporated and the residue was purified with flash column chromatography (hexane) to obtain a white solid **S3** (7.5 g, 86%). ^{1}H NMR (400 MHz, $CDCl_3$) δ 7.72 (t, *J* = 7.3 Hz, 2H), 7.41 (t, *J* = 7.7 Hz, 1H), 7.37 – 7.30 (m, 1H), 2.56 (s, 3H).

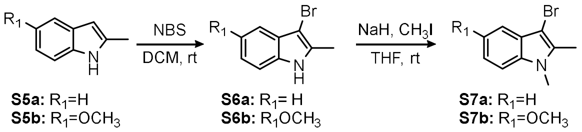

2-Methyl-1*H*-indole **S5a** (2.0 g, 15.25 mmol, 1eq) was dissolved in DCM containing silica gel (100mg) under N_2 atmosphere. NBS (2.71 g, 15.25 mmol, 1 eq) was added to the mixture and stirred for 30 min at room temperature. The reaction was quenched with water and extracted with DCM. The organic phase was dried with anhydrous Na_2SO_4 and evaporated under reduced pressure. The product **S6a** was directly used for the next step. NaH (913 mg, 60%, 22.85 mmol, 1.5 eq) was added to a solution of **S6a** in THF under N_2 atmosphere and stirred for 30 min at room temperature. Then iodomethane (3.24 g, 22.85 mmol, 1.5 eq) was added to the mixture and the reaction was stirred for 12 h at room temperature. The reaction was quenched with water and extracted with DCM. The organic phase was dried with anhydrous Na_2SO_4 and evaporated under reduced pressure. The residue was purified by column chromatography (EA: PE = 1: 10) to obtain a white solid **S7a** (2.8 g, 82%). ^{1}H NMR (400 MHz, $CDCl_3$) δ 7.49 (d, *J* = 7.6 Hz, 1H), 7.26 (d, *J* = 7.7 Hz, 1H), 7.23 – 7.18 (m, 1H), 7.15 (td, *J* = 7.3, 1.3 Hz, 1H), 3.70 (s, 3H), 2.45 (s, 3H).

3-Bromo-5-methoxy-1,2-dimethyl-1*H*-indole **S7b**. ^{1}H NMR (400 MHz, $CDCl_3$) δ 7.14 (d, *J* = 8.8 Hz, 1H), 6.93 (d, *J* = 2.3 Hz, 1H), 6.85 (dd, *J* = 8.8, 2.4 Hz, 1H), 3.88 (s, 3H), 3.67 (s, 3H), 2.42 (s, 3H).

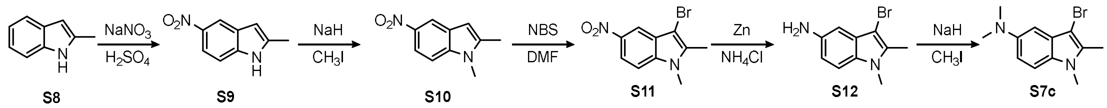

To a vigorously stirred solution of 2-methyl-1*H*-indole **S8** (1.0 g, 7.62 mmol, 1 eq) in concentrated H_2SO_4 at 0 ºC, a solution of $NaNO_3$ (712 mg, 8.39 mmol, 1.1 eq) in H_2SO_4 was added dropwise. The reaction was stirred for 10 min, and then poured into ice-water and precipitating a yellow solid. The product **S9** was isolated and washed with cold water. (1.17 g, 87%). ^{1}H NMR (400 MHz, $CDCl_3$) δ 8.46 (d, *J* = 2.2 Hz, 1H), 8.29 (s, 1H), 8.04 (dd, *J* = 8.9, 2.2 Hz, 1H), 7.30 (d, *J* = 8.9 Hz, 1H), 6.39 (s, 1H), 2.49 (s, 3H).

NaH (194 mg, 60%, 4.85 mmol, 1.5 eq) was added to a solution of 2-methyl-5-nitro-1*H*-indole **S9** (570 mg, 3.24 mmol, 1 eq) in THF under N_2 atmosphere and stirred for 30 min at room temperature. Then iodomethane (689 mg, 4.85 mmol, 1.5 eq) was added and the reaction was stirred for 12 h at room temperature. The reaction was quenched with water and extracted with DCM. The organic phase was dried with anhydrous Na_2SO_4 and evaporated under reduced pressure. The residue was purified by column chromatography (EA: PE = 1: 10) to obtain **S10** (500 mg, 81%). ^{1}H NMR (400 MHz, $CDCl_3$) δ 8.46 (d, *J* = 2.2 Hz, 1H), 8.06 (dd, *J* = 9.0, 2.2 Hz, 1H), 7.25 (d, *J* = 9.0 Hz, 1H), 6.43 (s, 1H), 3.72 (s, 3H), 2.46 (s, 3H).

NBS (1.97 g, 11.04 mmol, 1.05 eq) was added in batches to a solution of 1,2-dimethyl-5-nitro-1*H*-indole **S10** (2.0 g, 10.52 mmol, 1 eq) in DMF at 0 ºC and stirred for 1.5 h at room temperature. The reaction was poured over ice-water and precipitating crude product. The product was purified by column chromatography to get a yellow solid **S11** (2.0 g, 71%). ^{1}H NMR (400 MHz, $CDCl_3$) δ 8.44 (d, *J* = 2.3 Hz, 1H), 8.11 (dd, *J* = 9.0, 2.2 Hz, 1H), 7.28 (d, *J* = 9.0 Hz, 1H), 3.77 (s, 3H), 2.48 (s, 3H).

Zn power (1.21 g, 18.58 mmol, 10 eq) and NH_4Cl (994 mg, 18.58 mmol, 10 eq) were added to solution of 3-bromo-1,2-dimethyl-5-nitro-1*H*-indole **S11** (500 mg, 1.86 mmol, 1 eq) in THF/EtOH/H_2O (90 mL, v/v/v= 4: 3: 2) at 0 ºC. The reaction was stirred at 0 ºC for 10 min, warmed to room temperature

and then stirred for another 1 h. The reaction was evaporated under reduced pressure to remove THF and EtOH. Water was added to mixture to make the product precipitate out. The mixture was filtered to get a gray solid **S12** (405 mg, 91%). ^{1}H NMR (400 MHz, DMSO-*d6*) δ 7.13 (d, *J* = 8.5 Hz, 1H), 6.53 (dd, *J* = 8.6, 2.2 Hz, 1H), 6.50 (d, *J* = 2.1 Hz, 1H), 4.67 (s, 2H), 3.61 (s, 3H), 2.34 (s, 3H).

NaH (150 mg, 60%, 3.76 mmol, 2 eq) was added to a solution of 3-bromo-1,2-dimethyl-1*H*-indol-5-amine **S12** (450 mg, 1.88 mmol, 1 eq) in THF under N_2 atmosphere and stirred for 30 min at room temperature. Then iodomethane (534 mg, 3.76 mmol, 2 eq) was added to the mixture and the reaction was stirred for 12 h at room temperature. The reaction was quenched with water and extracted with DCM. The organic phase was dried with anhydrous Na_2SO_4 and evaporated under reduced pressure. The residue was purified by column chromatography (EA: PE = 1: 10) to obtain **S7c** (250 mg 50%). ^{1}H NMR (400 MHz, $CDCl_3$) δ 7.14 (d, *J* = 8.8 Hz, 1H), 6.87 (dd, *J* = 8.7, 2.3 Hz, 1H), 6.83 (d, *J* = 2.4 Hz, 1H), 3.65 (s, 3H), 2.95 (s, 6H), 2.41 (s, 3H).

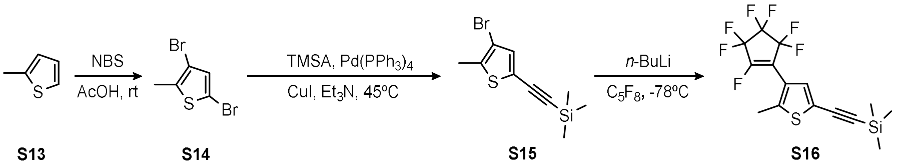

S13 **S14** **S15** **S16**

NBS (7.6 g, 42.79 mmol, 2.1 eq) was added to a solution of 2-methylthiophene **S13** (2.0 g, 20.37 mmol, 1 eq) in acetic acid (20 mL) at room temperature. The reaction was stirred for 2 h at room temperature. The solution was neutralized with aqueous NaOH and was extracted with CH_2Cl_2. The organic phase was dried with anhydrous Na_2SO_4 and evaporate to obtain the crude product. The crude product was purified by flash column chromatography (hexane) to gain the colorless liquid **S14** (3.82g, 73%). ^{1}H NMR (400 MHz, $CDCl_3$) δ 6.86 (s, 1H), 2.33 (s, 3H).

$Pd(PPh_3)_4$ (265 mg, 0.23 mmol, 0.05 eq), CuI (201 mg, 1.06 mmol, 0.23 eq) and TMSA (451 mg, 4.60 mmol, 1eq) were added to a solution of 3,5-dibromo-2-methylthiophene **S14** (3.53 g, 13.80 mmol, 3eq) in Et_3N (100 mL) under N_2 atmosphere. The reaction was stirred at 45 ºC for 7 h. The solvent was removed under reduced pressure. The residue was filtered through a short plug of silica gel, eluted with hexane. The organic solvent was evaporated and the residue was purified with flash column chromatography (hexane) to obtain a yellow solid **S15** (980 mg, 78%). ^{1}H NMR (400 MHz, $CDCl_3$) δ 7.02 (s, 1H), 2.36 (s, 3H), 0.23 (s, 9H).

((4-Bromo-5-methylthiophen-2-yl)ethynyl)trimethylsilane **S15** (1.17 g, 4.28 mmol, 1 eq) was dissolved in super-dry THF (30 mL) under N_2 atmosphere and then cooled to -78 ºC. *n*-Butyllithium (2.5 M, 1.71 mL, 1 eq) was added dropwise slowly and the mixture was stirred for 30 min at -78 ºC. Then perfluorocyclopentene (907 mg, 4.28 mmol, 1 eq) was added to the solution and stirred for 1 hour at -78 ºC. The reaction was quenched with water and extracted with DCM. The organic phase was dried with anhydrous Na_2SO_4. The organic solvent was evaporated and the residue was purified with flash column chromatography (hexane) to obtain a colorless liquid **S16** (980 mg, 59%). ^{1}H NMR (400 MHz, $CDCl_3$) δ 7.19 (s, 1H), 2.42 (s, 3H), 0.24 (s, 9H). ^{19}F NMR (376 MHz, $CDCl_3$) δ -108.55, -118.13, -126.73, -129.96.

Synthesis of monoacetylene

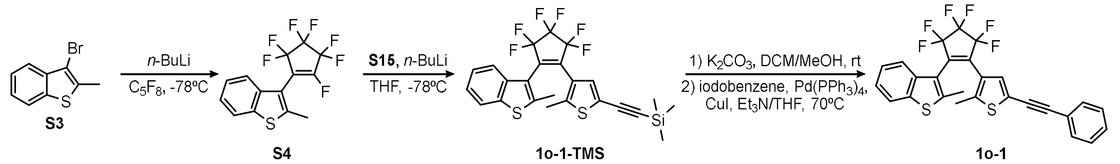

3-Bromo-2-methylbenzo[*b*]thiophene **S3** (200 mg, 0.88 mmol, 1 eq) was dissolved in super-dry THF (10 mL) under N_2 atmosphere and then cooled to -78 ºC. *n*-Butyllithium (2.5 M, 0.39 mL, 1.1 eq) was added dropwise slowly and the reaction was stirred for 30 min at -78 ºC. Then perfluorocyclopentene (205 mg, 0.97 mmol, 1.1 eq) was added and the reaction was stirred for 1 h at -78 ºC. The reaction was quenched with water and extracted with DCM. The organic phase was dried with anhydrous Na_2SO_4. The organic solvent was evaporated and the residue was purified with flash column chromatography (hexane) to obtain a white solid **S4** (167 mg, 56%). ^{1}H NMR (400 MHz, $CDCl_3$) δ 7.80 (d, *J* = 7.3 Hz, 1H), 7.48 (d, *J* = 7.8 Hz, 1H), 7.43 – 7.33 (m, 2H), 2.52 (s, 3H).

((4-Bromo-5-methylthiophen-2-yl)ethynyl)trimethylsilane **S15** (154 mg, 0.56 mmol, 1.1 eq) was dissolved in super-dry THF (10 mL) under N_2 atmosphere and then cooled to -78 ºC. *n*-Butyllithium (2.5 M, 0.25 mL, 1.2 eq) was added dropwise slowly and the mixture was stirred for 30 min at -78 ºC. Then 2-methyl-3-(perfluorocyclopent-1-en-1-yl)benzo[*b*]thiophene **S4** (174 mg, 0.51 mmol, 1 eq) was added to the solution and the reaction was stirred for 1 h at -78 ºC. The reaction was quenched with water and extracted with DCM. The organic phase was dried with anhydrous Na_2SO_4. The organic solvent was evaporated and the residue was purified with flash column chromatography (hexane) to obtain a white solid **1o-1-TMS** (116 mg, 44%). ^{1}H NMR (400 MHz, $CDCl_3$) δ 7.73 (d, *J* = 7.5 Hz, 1H), 7.53 (d, *J* = 7.5 Hz, 1H), 7.33 (m, 2H), 7.18 (s, 1H), 2.27 (s, 3H), 1.83 (s, 3H), 0.23 (s, 9H).

K_2CO_3 (20 mg, 0.15 mmol, 3 eq) was added to a solution of **1o-1-TMS** (25 mg, 0.05 mmol, 1 eq) in DCM/MeOH (2 mL, v/v = 1:1). The reaction was stirred for 30 min at room temperature. Water was added to the reaction and the reaction was extracted with DCM. The organic phase was dried with Na_2SO_4. The organic solvent was evaporated and was directly used for the next step. The crude solid, $Pd(PPh_3)_4$ (6 mg, 0.005 mmol, 0.1 eq) and CuI (3 mg, 0.015 mmol, 0.3 eq) were added to a two-necked flask. The flask was degassed and filled with N_2 for three times, and Et_3N/THF (5 mL, v/v = 1:1) was added with a syringe. Then iodobenzene (29 mg, 0.15 mmol, 3 eq) was added to the mixture and the reaction was stirred for 12 h at 70 ºC. The solvent was removed under reduced pressure. The residue was filter through a short plug of silica gel, eluted with hexane. The organic solvent was evaporated and the residue was purified with flash column chromatography (hexane) to obtain a white solid **1o-1** (8 mg, 32%). ^{1}H NMR (400 MHz, $CDCl_3$) δ 7.77 (d, *J* = 7.0 Hz, 1H), 7.57 (d, *J* = 7.2 Hz, 1H), 7.50 (dd, *J* = 6.7, 3.1 Hz, 2H), 7.43 – 7.30 (m, 5H), 7.23 (s, 1H), 2.32 (s, 3H), 1.90 (s, 3H). ^{19}F NMR (376 MHz, $CDCl_3$) δ -109.40, -109.67, -110.11, -111.66, -112.37, -131.50, -132.14, -133.40, -134.04. HRMS (ESI): calcd for $C_{27}H_{16}F_6S_2^+$ $[M]^+$ 518.0592, found 518.0645.

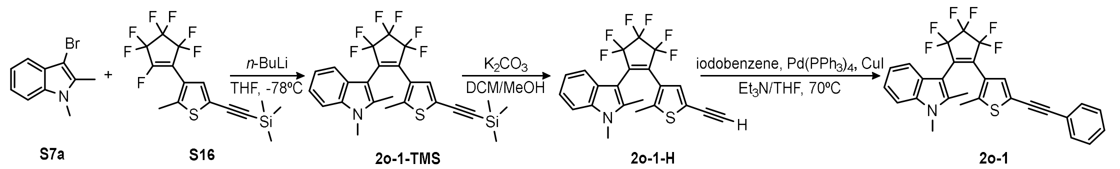

3-Bromo-1,2-dimethyl-1*H*-indole **S7a** (500 mg, 2.23 mmol, 2 eq) was dissolved in super-dry THF (20 mL) under N_2 atmosphere and then cooled to -78 ºC. *n*-Butyllithium (2.5 M, 0.89 mL, 2 eq) was added dropwise slowly and the reaction was stirred for 30 min at -78 ºC. Then a solution of **S16** (431

mg, 1.12 mmol, 1 eq) in super-dry THF (1 mL) was added and the reaction was stirred for 1 h at -78 ºC. The reaction was quenched with water and extracted with EA. The organic phase was dried with anhydrous Na_2SO_4. The organic solvent was evaporated and the residue was purified with flash column chromatography (EA: PE= 1: 10) to obtain a little green solid (480 mg, 84%) and recrystallized by DCM/hexane to afford a yellow crystalline solid **2o-1-TMS**. ^1H NMR (400 MHz, DMSO-*d6*) δ 7.51 (d, *J* = 8.2 Hz, 1H), 7.44 (s, 1H), 7.40 (d, *J* = 8.0 Hz, 1H), 7.23 – 7.17 (m, 1H), 7.13 – 7.06 (m, 1H), 3.69 (s, 3H), 2.03 (s, 3H), 1.71 (s, 3H), 0.23 (s, 9H). ^{19}F NMR (376 MHz, $CDCl_3$) δ -110.07, -132.48. HRMS (ESI): calcd for $C_{25}H_{23}F_6NSSi^+$ $[M]^+$ 511.1219, found 511.1218.

K_2CO_3 (81mg, 0.59 mmol, 3 eq) was added to a solution of **2o-1-TMS** (100 mg, 0.20 mmol, 1 eq) in DCM/MeOH (5 mL, v/ v=1: 1) at room temperature. The reaction was stirred for 30 min at room temperature. The product was purified by column chromatography (EA: PE= 1: 10) to get a green solid and recrystallized by DCM/hexane to afford a light green crystalline solid **2o-1-H**. (83 mg, 97%).^1H NMR (400 MHz, $CDCl_3$) δ 7.56 (d, *J* = 8.0 Hz, 1H), 7.32 (s, 1H), 7.28 (d, *J* = 8.1 Hz, 1H), 7.25 – 7.20 (m, 1H), 7.15 (td, *J* = 7.5, 1.3 Hz, 1H), 3.64 (s, 3H), 3.33 (s, 1H), 1.99 (s, 3H), 1.74 (s, 3H). ^{19}F NMR (376 MHz, $CDCl_3$) δ-110.05, -132.49. HRMS (ESI): calcd for $C_{22}H_{15}F_6NS^+$ $[M]^+$ 439.0824, found 439.0820.

2o-1-H (20 mg, 0.05 mmol, 1 eq), $Pd(PPh_3)_4$ (5 mg, 0.005mmol, 0.1 eq) and CuI (3 mg, 0.013 mmol, 0.3 eq) were added to a two-necked flask. The flask was degassed and filled with N_2 for three times. Et_3N/THF (10 mL, v/v = 1:1) was added by using the syringe. Then iodobenzene (28 mg, 0.14 mmol, 3 eq) was added to the mixture and the reaction was stirred for 12 h at 70 ºC. The solvent was removed under reduced pressure. The residue was filtered through a short plug of silica gel, eluted with hexane. The organic solvent was evaporated and the residue was purified with flash column chromatography (EA: PE= 1: 10) to obtain a green solid and recrystallized by DCM/hexane to afford a light green crystalline solid **2o-1** (15 mg, 64%). ^1H NMR (400 MHz, $CDCl_3$) δ 7.59 (d, *J* = 8.0 Hz, 1H), 7.50 (dd, *J* = 6.6, 3.0 Hz, 2H), 7.38 – 7.33 (m, 3H), 7.33 (s, 1H), 7.28 (d, *J* = 8.2 Hz, 1H), 7.23 (t, *J* = 7.5 Hz, 1H), 7.16 (t, *J* = 7.4 Hz, 1H), 3.65 (s, 3H), 2.02 (s, 3H), 1.76 (s, 3H). ^{19}F NMR (376 MHz, $CDCl_3$) δ -109.98, -132.48. HRMS (ESI): calcd for $C_{28}H_{19}F_6NS^+$ $[M]^+$ 515.1137, found 515.1130.

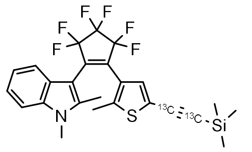

2o-1-13C-TMS

2o-1-^{13}C-TMS. ^1H NMR (400 MHz, $CDCl_3$) δ 7.57 (d, *J* = 7.9 Hz, 1H), 7.29 (s, 1H), 7.28 (s, 1H), 7.25 – 7.20 (m, 1H), 7.15 (ddd, *J* = 8.1, 7.0, 1.3 Hz, 1H), 3.64 (s, 3H), 1.98 (s, 3H), 1.72 (s, 3H), 0.24 (d, *J* = 2.6 Hz, 9H). ^{19}F NMR (376 MHz, $CDCl_3$) δ -110.07, -132.51. ^{13}C NMR (101 MHz, $CDCl_3$) δ 98.84, 97.16. HRMS (ESI): calcd for $C_{23}{}^{13}C_2H_{23}F_6NSSi^+$ $[M]^+$ 513.1280, found 513.1286.

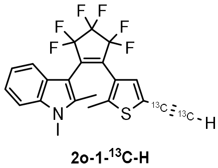

2o-1-13C-H

2o-1-^{13}C-H. ^1H NMR (400 MHz, $CDCl_3$) δ 7.56 (d, *J* = 8.0 Hz, 1H), 7.31 (d, *J* = 4.0 Hz, 1H), 7.28 (d, *J* = 8.2 Hz, 1H), 7.23 (td, *J* = 6.9, 1.2 Hz, 1H), 7.14 (ddd, *J* = 8.1, 7.0, 1.3 Hz, 1H), 3.64 (s, 3H), 3.32 (dd, *J* = 248, 56 Hz, 1H), 1.99 (s, 3H), 1.74 (s, 3H). ^{19}F NMR (376 MHz, $CDCl_3$) δ -110.05, -132.51. ^{13}C

NMR (101 MHz, $CDCl_3$) δ 82.74, 80.92, 76.90, 75.09. HRMS (ESI): calcd for $C_{20}{}^{13}C_2H_{15}F_6NS^+$ $[M]^+$ 441.0890, found 441.0892.

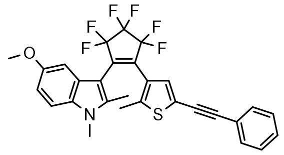

3o-1

3o-1. ^{1}H NMR (400 MHz, $CDCl_3$) δ 7.53 – 7.46 (m, 2H), 7.35 (m, 4H), 7.16 (d, *J* = 8.9 Hz, 1H), 6.96 (d, *J* = 2.4 Hz, 1H), 6.86 (dd, *J* = 8.9, 2.4 Hz, 1H), 3.82 (s, 3H), 3.62 (s, 3H), 2.03 (s, 3H), 1.75 (s, 3H). ^{19}F NMR (376 MHz, $CDCl_3$) δ -109.76, -132.48. HRMS (ESI): calcd for $C_{29}H_{21}F_6NOS^+$ $[M]^+$ 545.1243, found 545.1246.

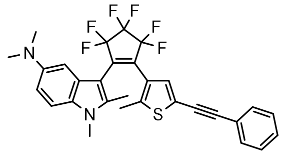

4o-1

4o-1. ^{1}H NMR (400 MHz, $CDCl_3$) δ 7.49 (dd, *J* = 6.7, 3.0 Hz, 2H), 7.37 (s, 1H), 7.36 – 7.32 (m, 3H), 7.15 (d, *J* = 8.8 Hz, 1H), 6.85 (m, 2H), 3.60 (s, 3H), 2.91 (s, 6H), 2.03 (s, 3H), 1.76 (s, 3H). ^{19}F NMR (376 MHz, $CDCl_3$) δ -109.52, -132.67. HRMS (ESI): calcd for $C_{30}H_{25}F_6N_2S^+$ $[M+H]^+$ 559.1637, found 559.1637.

Synthesis of diyne

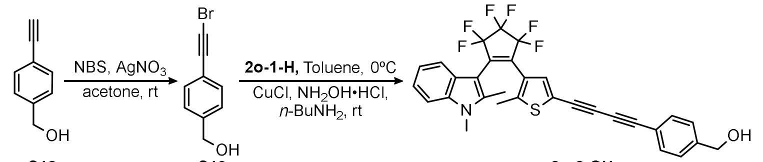

S18 S19 2o-2-OH

NBS (141mg, 0.79 mmol, 1.05 eq) was added to a solution of (4-ethynylphenyl)methanol **S18** (100 mg, 0.76 mmol, 1 eq) in acetone at room temperature. $AgNO_3$ (12.85 mg, 0.08 mmol, 0.1 eq) was added and the reaction was stirred for 2 h at room temperature. The product was purified by column chromatography (EA: PE= 1: 3) to get a white solid **S19** (150 mg, 94%). ^{1}H NMR (400 MHz, $CDCl_3$) δ 7.45 (d, *J* = 8.3 Hz, 2H), 7.31 (d, *J* = 8.5 Hz, 2H), 4.70 (s, 2H).

CuCl (4 mg, 0.04 mmol, 0.33 eq), $NH_2OH{\cdot}HCl$ (5 mg, 0.08 mmol, 0.66 eq) and *n*-$BuNH_2$ (33 mg, 0.46 mmol, 4 eq) were added in order to a solution of **2o-1-H** (50 mg, 0.11 mmol, 1 eq) in toluene at 0 °C. The solution was stirred for 5 min and (4-(bromoethynyl)phenyl)methanol **S19** (72 mg, 0.34 mmol, 3 eq) was added dropwise to the mixture at 0 °C. The reaction was warmed to room temperature slowly and stirred overnight. The reaction was quenched with water and extracted with DCM. The organic phase was dried with anhydrous Na_2SO_4 and evaporated under reduced pressure. The residue was purified with column chromatography (EA: PE= 1: 3) to get a light green solid **2o-2-OH** (50 mg, 77%). ^{1}H NMR (400 MHz, $CDCl_3$) δ 7.55 (d, *J* = 7.9 Hz, 1H), 7.51 (d, *J* = 7.9 Hz, 2H), 7.37 (d, *J* = 5.9 Hz, 2H), 7.34 (s, 1H), 7.28 (d, *J* = 8.0 Hz, 1H), 7.23 (t, *J* = 7.5 Hz, 1H), 7.15 (t, *J* = 7.4 Hz, 1H), 4.73 (s, 2H), 3.65 (s, 3H), 2.00 (s, 3H), 1.77 (s, 3H), 1.69 (s, 1H). ^{19}F NMR (376 MHz, $CDCl_3$) δ -109.94, -132.47. HRMS (ESI): calcd for $C_{31}H_{22}F_6NOS^+$ $[M+H]^+$ 570.1321, found 570.1328.

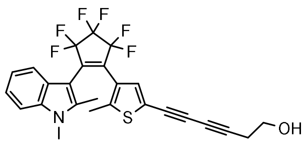

2-2-$(CH_2)_2$OH

2-2-$(CH_2)_2$OH. ^{1}H NMR (400 MHz, $CDCl_3$) δ 7.54 (d, *J* = 7.9 Hz, 1H), 7.32 (s, 1H), 7.29 (d, *J* = 7.9 Hz, 1H), 7.23 (t, *J* = 7.4 Hz, 1H), 7.14 (t, *J* = 7.4 Hz, 1H), 3.85 (s, 2H), 3.64 (s, 3H), 2.67 (t, *J* = 5.3 Hz, 2H), 1.98 (s, 3H), 1.75 (s, 3H). ^{19}F NMR (376 MHz, $CDCl_3$) δ -109.97, -132.47. HRMS (ESI): calcd for $C_{26}H_{18}F_6NOS^-$ $[M-H]^-$ 506.1019, found 506.1018.

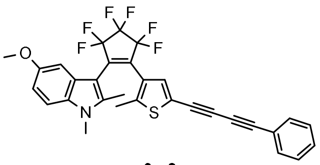

3o-2

3o-2. ^{1}H NMR (400 MHz, $CDCl_3$) δ 7.55 – 7.48 (m, 2H), 7.41 (s, 1H), 7.41 – 7.30 (m, 3H), 7.16 (d, *J* = 8.8 Hz, 1H), 6.93 (d, *J* = 2.4 Hz, 1H), 6.86 (dd, *J* = 8.9, 2.4 Hz, 1H), 3.81 (s, 3H), 3.62 (s, 3H), 2.01 (s, 3H), 1.76 (s, 3H). ^{19}F NMR (376 MHz, $CDCl_3$) δ -109.76, -132.48. HRMS (ESI): calcd for $C_{31}H_{21}F_6NOS^+$ $[M]^+$ 569.1248, found 569.1240.

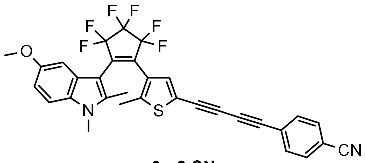

3o-2-CN

3o-2-CN. ^{1}H NMR (400 MHz, $CDCl_3$) δ 7.66 – 7.56 (m, 4H), 7.45 (s, 1H), 7.16 (d, *J* = 8.8 Hz, 1H), 6.91 (d, *J* = 2.4 Hz, 1H), 6.86 (dd, *J* = 8.8, 2.4 Hz, 1H), 3.80 (s, 3H), 3.62 (s, 3H), 2.02 (s, 3H), 1.78 (s, 3H). ^{19}F NMR (376 MHz, $CDCl_3$) δ -109.67, -132.45. HRMS (ESI): calcd for $C_{32}H_{20}F_6N_2OS^+$ $[M]^+$ 594.1195, found 594.1196.

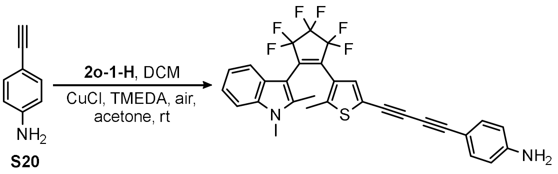

2o-2-NH_2

A solution of TMEDA (8 mg, 0.07 mmol, 2 eq) and CuCl (3 mg, 0.03 mmol, 1 eq) in acetone were stirred for 10 min with air bubbled at room temperature. A solution of 4-ethynylaniline **S20** (20 mg, 0.17 mmol, 5 eq) and **2o-1-H** (15 mg, 0.03 mmol, 1 eq) in DCM was added and the reaction was stirred for 1 h at room temperature. The product was purified with column chromatography (EA: PE= 1: 3) to get a light yellow solid **2o-2-NH_2** (10 mg, 53%). ^{1}H NMR (400 MHz, $CDCl_3$) δ 7.56 (d, *J* = 8.0 Hz, 1H), 7.33 (d, *J* = 2.2 Hz, 2H), 7.31 (s, 1H), 7.28 (d, *J* = 7.9 Hz, 1H), 7.23 (t, *J* = 7.3 Hz, 1H), 7.15 (t, *J* = 7.5 Hz, 1H), 6.60 (d, *J* = 8.6 Hz, 2H), 3.65 (s, 3H), 1.99 (s, 3H), 1.76 (s, 3H). ^{19}F NMR (376 MHz, $CDCl_3$) δ -109.97, -132.48. HRMS (ESI): calcd for $C_{30}H_{21}F_6N_2S^+$ $[M+H]^+$ 555.1324, found 555.1324.

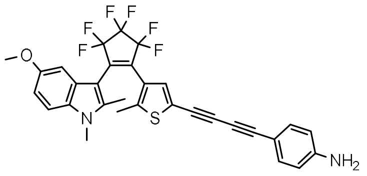

3o-2-NH$_2$

3o-2-NH$_2$. ^{1}H NMR (400 MHz, CDCl$_3$) δ 7.36 (s, 1H), 7.32 (d, *J* = 8.5 Hz, 2H), 7.16 (d, *J* = 8.9 Hz, 1H), 6.93 (d, *J* = 2.4 Hz, 1H), 6.86 (dd, *J* = 8.9, 2.4 Hz, 1H), 6.60 (d, *J* = 8.6 Hz, 2H), 3.98 (s, 2H), 3.81 (s, 3H), 3.62 (s, 3H), 2.01 (s, 3H), 1.75 (s, 3H). ^{19}F NMR (376 MHz, CDCl$_3$) δ -109.72, -132.46. HRMS (ESI): calcd for $C_{31}H_{23}F_6N_2OS^+$ $[M+H]^+$ 585.1430, found 585.1430.

Synthesis of Triyne

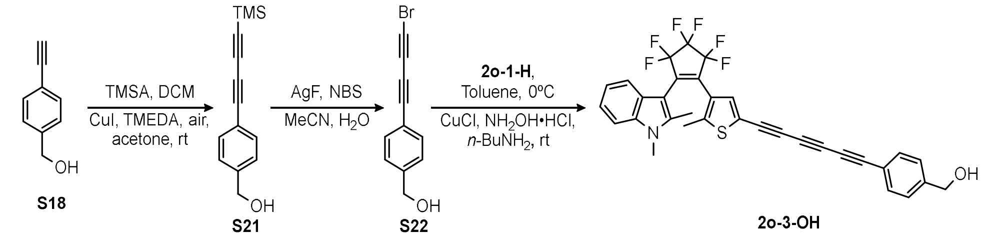

A solution of TMEDA (351 mg, 3.03 mmol, 2 eq) and CuCl (150 mg, 1.51 mmol, 1 eq) in acetone was stirred for 10 min with air bubbled at room temperature. A solution containing (4-ethynylphenyl)methanol **S18** (200 mg, 1.51 mmol, 1 eq) and TMSA (743 mg, 7.57 mmol, 5 eq) in DCM was added and the reaction was stirred for 1 h at room temperature. The product was purified with column chromatography (EA: PE= 1: 3) to get a light yellow solid **S21** (290 mg, 84%). ^{1}H NMR (400 MHz, CDCl$_3$) δ 7.48 (d, *J* = 8.2 Hz, 2H), 7.32 (d, *J* = 8.2 Hz, 2H), 4.71 (d, *J* = 5.4 Hz, 2H), 1.68 (t, *J* = 6.4 Hz, 1H), 0.23 (s, 9H).

AgF (161 mg, 1.27 mmol, 1 eq) was added to a solution of **S21** (290 mg, 1.27 mmol, 1 eq) in acetonitrile containing H_2O (18 μL). The mixture was stirred for 30 min at room temperature. NBS (226 mg, 1.27 mmol, 1 eq) was added to the mixture for 3 h at room temperature. The product was purified with column chromatography (EA: PE= 1: 3) to obtain a yellow oil **S22** (140 mg, 47%). ^{1}H NMR (400 MHz, CDCl$_3$) δ 7.50 (d, *J* = 8.2 Hz, 2H), 7.33 (d, *J* = 8.1 Hz, 2H), 4.71 (s, 2H), 1.67 (s, 1H).

2o-3-OH was synthesized according to the method of **2o-2-OH**. The product was a green solid and recrystallized by DCM/hexane to afford a light green crystalline solid. ^{1}H NMR (400 MHz, CDCl$_3$) δ 7.58 – 7.50 (m, 3H), 7.42 (s, 1H), 7.35 (d, *J* = 8.0 Hz, 2H), 7.28 (d, *J* = 8.1 Hz, 1H), 7.25 – 7.20 (m, 1H), 7.15 (ddd, *J* = 8.0, 6.9, 1.3 Hz, 1H), 4.73 (s, 2H), 3.65 (s, 3H), 2.00 (s, 3H), 1.77 (s, 3H). ^{19}F NMR (376 MHz, CDCl$_3$) δ -109.93, -132.46. HRMS (ESI): calcd for $C_{33}H_{22}F_6NOS^+$ $[M+H]^+$ 594.1321, found 594.1328.

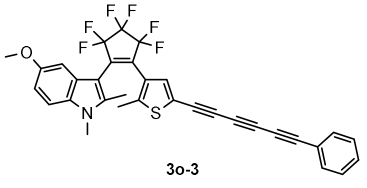

3o-3

3o-3. ^{1}H NMR (400 MHz, CDCl$_3$) δ 7.57 – 7.51 (m, 2H), 7.45 (s, 1H), 7.43 – 7.38 (m, 1H), 7.34 (m, 2H), 7.16 (d, *J* = 8.9 Hz, 1H), 6.92 (d, *J* = 2.3 Hz, 1H), 6.86 (dd, *J* = 8.8, 2.4 Hz, 1H), 3.80 (s, 3H), 3.62 (s, 3H), 2.01 (s, 3H), 1.76 (s, 3H). ^{19}F NMR (376 MHz, CDCl$_3$) δ -109.71, -132.51. HRMS (ESI): calcd for $C_{33}H_{22}F_6NOS^+$ $[M+H]^+$ 594.1321, found 594.1367

Synthesis of Tetrayne

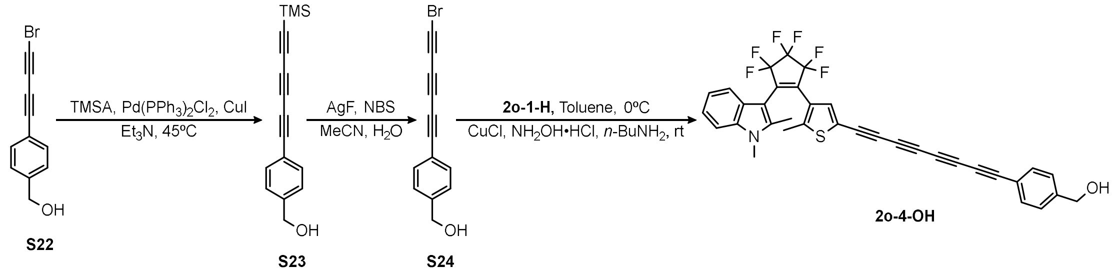

$PdCl_2(PPh_3)_2$ (64 mg, 0.09 mmol, 0.1 eq) and CuI (17 mg, 0.09 mmol, 0.1 eq) were added to a two-necked flask. The flask was degassed and filled with N_2 for three times. A solution of **S22** (215 mg, 0.91 mmol, 1 eq) and TMSA (180 mg, 1.83 mmol, 2 eq) in Et_3N (10 mL) was added with a syringe and the reaction was stirred for 2 h at 50 ºC. The solvent was removed under reduced pressure. The residue was purified with flash column chromatography (EA: PE= 1: 3) to obtain a yellow oil **S23** (70 mg, 30%). ^1H NMR (400 MHz, $CDCl_3$) δ 7.52 (d, J = 8.3 Hz, 2H), 7.33 (d, J = 8.0 Hz, 2H), 4.72 (s, 2H), 1.69 (s, 1H), 0.22 (s, 9H).

S24 was synthesized according to the method of **S22**. ^1H NMR (400 MHz, $CDCl_3$) δ 7.52 (d, J = 8.2 Hz, 2H), 7.34 (d, J = 8.1 Hz, 2H), 4.72 (s, 2H).

2o-4-OH was synthesized according to the method of **2o-2-OH**. The product was a green solid and recrystallized by DCM/hexane to afford a light green crystalline solid. ^1H NMR (400 MHz, $CDCl_3$) δ 7.54 (d, J = 8.0 Hz, 3H), 7.45 (s, 1H), 7.35 (d, J = 8.1 Hz, 2H), 7.28 (d, J = 8.0 Hz, 1H), 7.22 (d, J = 7.9 Hz, 1H), 7.14 (t, J = 7.2 Hz, 1H), 4.73 (s, 2H), 3.65 (s, 3H), 1.99 (s, 3H), 1.77 (s, 3H). ^{19}F NMR (376 MHz, $CDCl_3$) δ -109.91, -132.45. HRMS (ESI): calcd for $C_{35}H_{20}F_6NOS^-$ $[M-H]^-$ 616.1175, found 616.1178.

Synthesis of Mito-switch

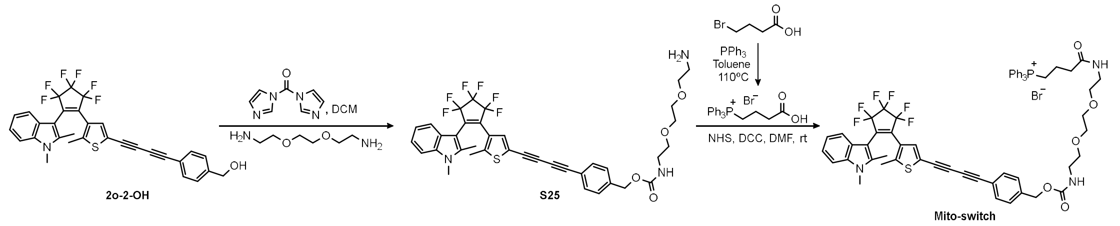

4-bromobutanoic acid (1.27 g, 7.63 mmol, 1 eq) and triphenylphosphine (2.0 g, 7.63 mmol, 1 eq) were dissolved in toluene and stirred at 110 ºC overnight. A white solid was generated and the mixture were filtered to obtain (3-Carboxypropyl)triphenylphosphonium bromide (2.45 g, 75%). ^1H NMR (400 MHz, DMSO-*d6*) δ 12.35 (s, 1H), 7.95 – 7.68 (m, 15H), 3.62 – 3.49 (m, 2H), 2.47 (t, J = 7.0 Hz, 2H), 1.73 (q, J = 7.9 Hz, 2H).

Di(1*H*-imidazol-1-yl)methanone (28 mg, 0.18 mmol, 2 eq) was added to a solution of **2o-2-OH** (50 mg, 0.09 mmol, 1eq) in DCM (2 mL) and the mixture was stirred at room temperature for 6 h. The reaction was quenched with water and extracted with DCM. The organic phase was dried with anhydrous Na_2SO_4 and evaporated under reduced pressure. The residue was dissolved in DCM (1 mL), and 2,2'-(ethane-1,2-diylbis(oxy))bis(ethan-1-amine) (245 mg, 0.44 mmol, 5 eq) was added to the mixture. The reaction was stirred at room temperature for 20 h. The residue was filter through a short plug of silica gel eluted with DCM/ MeOH (v/ v= 10:1) to obtain **S25**. (3-Carboxypropyl)triphenylphosphonium

bromide (250 mg, 0.58 mmol, 6 eq), dicyclohexylcarbodiimide (DCC, 144 mg, 0.70 mmol, 7.2 eq) and *N*-hydroxysuccinimide (NHS, 100 mg, 0.87 mmol, 9 eq) were dissolved in anhydrous DMF at 0 °C under N_2 atmosphere and stirred for 8 h at rt. The mixture was filtrated and added to a solution of **S25** in anhydrous DMF (1 mL). The reaction was stirred for overnight at room temperature. The reaction was quenched with water and extracted with DCM. The organic phase was dried with anhydrous Na_2SO_4 and evaporated under reduced pressure. The residue was purified with column chromatography (DCM: MeOH= 10: 1) to get a green solid **Mito-switch** (34 mg, 32%). ^{1}H NMR (400 MHz, $CDCl_3$) 7.80 - 7.73 (m, 9H), 7.70 - 7.65 (m, 6H), 7.55 (d, *J* = 8.0 Hz, 1H), 7.41 (d, *J* = 7.9 Hz, 2H), 7.36 (s, 1H), 7.31 (d, *J* = 8.3 Hz, 2H), 7.27 (d, *J* = 8.3 Hz, 1H), 7.25 – 7.20 (m, 1H), 7.14 (dd, *J* = 8.1, 6.8 Hz, 1H), 5.12 (s, 1H), 5.06 (s, 2H), 3.75 - 3.67 (m, 2H), 3.65 (s, 3H), 3.63 – 3.59 (m, 4H), 3.58 – 3.54 (m, 4H), 3.46 (q, *J* = 5.4 Hz, 2H), 3.38 (q, *J* = 5.4 Hz, 2H), 2.75 (t, *J* = 6.2 Hz, 2H), 2.00 (s, 3H), 1.95 – 1.89 (m, 2H), 1.77 (s, 3H). ^{19}F NMR (376 MHz, $CDCl_3$) δ -109.96, -132.49. HRMS (ESI): calcd for $C_{60}H_{55}F_6N_3O_5PS^+$ [M]$^+$ 1074.3499, found 1074.3497.

Synthesis of Lyso-switch

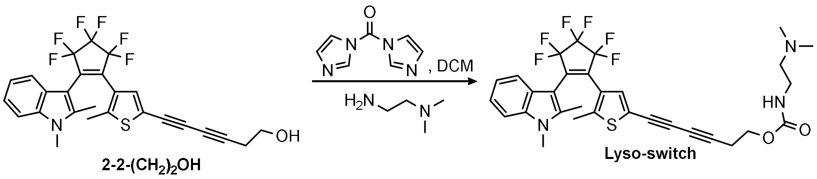

Di(1*H*-imidazol-1-yl)methanone (8 mg, 0.05 mmol, 2 eq) was added to a solution of **2-2-(CH$_2$)$_2$OH** (13 mg, 0.03 mmol, 1 eq) in DCM (2 mL) and stirred at room temperature for 6 h. Water was added and the mixture was extracted with DCM. The organic phase was dried with anhydrous Na_2SO_4 and evaporated under reduced pressure. The residue was directly used for the next step. The residue was dissolved in DCM (1 mL) and N^1,N^1-dimethylethane-1,2-diamine (5 mg, 0.05 mmol, 2 eq) was added to the mixture and stirred at room temperature for 20 h. The product was purified with column chromatography (DCM: MeOH= 10: 1) to get a yellow solid and recrystallized by DCM/hexane to afford a light green crystalline solid **Lyso-switch** (6 mg, 37%). ^{1}H NMR (400 MHz, $CDCl_3$) δ 7.54 (d, *J* = 7.9 Hz, 1H), 7.32 (s, 1H), 7.29 (s, 1H), 7.22 (t, *J* = 7.5 Hz, 1H), 7.14 (t, *J* = 7.3 Hz, 1H), 5.69 (s, 1H), 4.20 (t, *J* = 6.7 Hz, 2H), 3.64 (s, 3H), 3.40 (d, *J* = 6.2 Hz, 2H), 2.71 (t, *J* = 6.7 Hz, 2H), 2.66 (s, 2H), 2.43 (s, 6H), 1.98 (s, 3H), 1.74 (s, 3H). ^{19}F NMR (376 MHz, $CDCl_3$) δ -109.97, -132.53. HRMS (ESI): calcd for $C_{31}H_{30}F_6N_3O_2S^+$ [M+H]$^+$ 622.1957, found 622.1959.

Synthesis of PM-switch

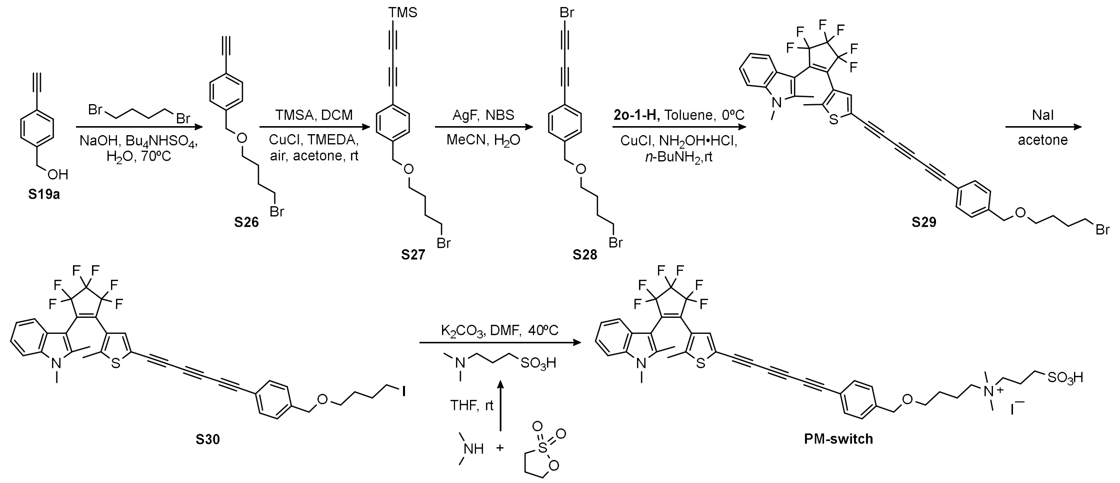

S19a (300 mg, 2.27 mmol, 1 eq), 1,4-dibromobutane (1.23 g, 5.67 mmol, 2.5 eq) and tetrabutylammonium hydrogen sulfate (385 mg, 1.13 mmol, 0.5 eq) were added to 10% aqueous NaOH and stirred at 70 ºC for 5 h. The product was extracted with DCM. The organic phase was dried with anhydrous Na_2SO_4 and evaporated under reduced pressure. The residue was purified with column chromatography (PE: EA= 20: 1) to obtain a colorless oil **S26** (405 mg, 67%). ^1H NMR (400 MHz, $CDCl_3$) δ 7.47 (d, *J* = 8.2 Hz, 2H), 7.28 (d, *J* = 8.3 Hz, 2H), 4.50 (s, 2H), 3.50 (t, *J* = 6.2 Hz, 2H), 3.44 (t, *J* = 6.7 Hz, 2H), 3.06 (s, 1H), 1.98 (dt, *J* = 14.5, 6.8 Hz, 2H), 1.77 (dt, *J* = 13.2, 6.3 Hz, 2H).

S27 was synthesized according to the method of **S21**. ^1H NMR (400 MHz, $CDCl_3$) δ 7.49 (d, *J* = 8.2 Hz, 2H), 7.30 (d, *J* = 8.0 Hz, 2H), 4.51 (s, 2H), 3.53 (t, *J* = 6.2 Hz, 2H), 3.46 (t, *J* = 6.7 Hz, 2H), 2.00 (dt, *J* = 14.5, 6.9 Hz, 2H), 1.83 – 1.75 (m, 2H), 0.26 (s, 9H).

S28 was synthesized according to the method of **S22.** ^1H NMR (400 MHz, $CDCl_3$) δ 7.50 (d, *J* = 8.2 Hz, 2H), 7.30 (d, *J* = 8.2 Hz, 2H), 4.52 (s, 2H), 3.53 (t, *J* = 6.2 Hz, 2H), 3.46 (t, *J* = 6.7 Hz, 2H), 2.00 (dt, *J* = 14.4, 6.8 Hz, 2H), 1.83 – 1.75 (m, 2H).

S29 was synthesized according to the method of **2o-2-OH**. ^1H NMR (400 MHz, $CDCl_3$) δ 7.52 (m, 3H), 7.42 (s, 1H), 7.32 – 7.27 (m, 3H), 7.23 (t, *J* = 7.3 Hz, 1H), 7.15 (t, *J* = 7.6 Hz, 1H), 4.51 (s, 2H), 3.65 (s, 3H), 3.51 (t, *J* = 6.2 Hz, 2H), 3.44 (t, *J* = 6.7 Hz, 2H), 1.98 (m, 5H), 1.81 – 1.74 (m, 5H).

S29 (40 mg, 0.05 mmol, 1 eq) and NaI (82 mg, 0.55 mmol, 10 eq) were dissolved in acetone and stirred at room temperature overnight. Water was added to the mixture was extracted with DCM. The organic phase was dried with anhydrous Na_2SO_4 and evaporated under reduced pressure. The residue was purified with column chromatography (PE: EA= 10: 1) to obtain a green solid **S30** (35 mg, 82%). ^1H NMR (400 MHz, $CDCl_3$) δ 7.58 – 7.48 (m, 3H), 7.42 (s, 1H), 7.28 (d, *J* = 13.5 Hz, 3H), 7.27 – 7.19 (m, 1H), 7.15 (ddd, *J* = 8.0, 6.9, 1.3 Hz, 1H), 4.50 (s, 2H), 3.65 (s, 3H), 3.50 (t, *J* = 6.1 Hz, 2H), 3.22 (t, *J* = 6.9 Hz, 2H), 2.00 (s, 3H), 1.95 (m, 2H), 1.77 (s, 3H), 1.73 (dt, *J* = 6.2, 3.7 Hz, 2H).

1,2-Oxathiolane 2,2-dioxide (250 mg, 2.05 mmol, 1 eq) was dissolved in THF and Dimethylamine (2M in MeOH, 10 mL, 10eq) was added to the mixture and the reaction was stirred at room temperature overnight. The solution was evaporated under reduced pressure and concentrated to 10 mL. 15 mL of diethyl ether was added to the mixture, generating a gray solid. The solid was filtered to obtain 3-(dimethylamino)propane-1-sulfonic acid (305 mg, 89%). ^1H NMR (400 MHz, MeOD) δ 3.30 (s, 2H), 2.94 (t, *J* = 6.8 Hz, 2H), 2.91 (s, 6H), 2.19 (dt, *J* = 13.6, 7.1 Hz, 2H).

S30 (10 mg, 0.01 mmol, 1 eq), 3-(dimethylamino)propane-1-sulfonic acid (6 mg, 0.04 mmol, 3 eq) and K_2CO_3 (18 mg, 0.13 mmol, 10 eq) were suspended in DMF/ DMSO (1 mL, v/ v= 1: 1) and stirred at 45 ºC for 12 h. Water was added and the mixture was extracted with DCM. The organic phase was dried

with anhydrous Na_2SO_4 and evaporated under reduced pressure. The product was purified with C18 reversed-phase column chromatography to obtain a light green solid **PM-switch** (3 mg, 25%). ^{1}H NMR (400 MHz, MeOD) δ 7.58 (d, J = 8.2 Hz, 2H), 7.54 (s, 1H), 7.45 – 7.41 (m, 3H), 7.35 (d, J = 8.1 Hz, 1H), 7.23 (t, J = 7.7 Hz, 1H), 7.12 (t, J = 7.5 Hz, 1H), 4.58 (s, 2H), 3.72 (s, 3H), 3.66 (t, J = 2.7 Hz, 2H), 3.61 (t, J = 6.0 Hz, 2H), 3.53 (t, J = 3.9 Hz, 2H), 3.10 (s, 6H), 2.88 (t, J = 6.8 Hz, 2H), 2.25 – 2.17 (m, 2H), 2.09 (s, 3H), 1.92 (m, 2H), 1.78 (s, 3H), 1.72 (m, 2H). ^{19}F NMR (376 MHz, $CDCl_3$) δ -109.86, -132.44. HRMS (ESI): calcd for $C_{42}H_{41}F_6N_2O_4S_2^+$ $[M]^+$ 815.2406, found 815.2409.

Supplementary References

1 Fredrich, S., Bonasera, A., Valderrey, V. & Hecht, S. Sensitive Assays by Nucleophile-Induced Rearrangement of Photoactivated Diarylethenes. *J Am Chem Soc* **140**, 6432-6440 (2018).

2 Hu, F. *et al.* Supermultiplexed optical imaging and barcoding with engineered polyynes. *Nat Methods* **15**, 194-200 (2018).
